# Refinement of anomalous dispersion correction parameters in single-crystal structure determinations

**DOI:** 10.1107/S2052252522006844

**Published:** 2022-07-20

**Authors:** Florian Meurer, Oleg V. Dolomanov, Christoph Hennig, Norbert Peyerimhoff, Florian Kleemiss, Horst Puschmann, Michael Bodensteiner

**Affiliations:** a Faculty for Chemistry and Pharmacy, University of Regensburg, Universitätsstrasse 31, Regensburg 93053, Germany; b OlexSys Ltd, Chemistry Department, Durham University, Durham DH1 3LE, United Kingdom; c Institute of Resource Ecology, Helmholtz-Zentrum Dresden-Rossendorf (HZDR), Bautzner Landstrasse 400, Dresden 01314, Germany; d Rossendorf Beamline (BM20-CRG), European Synchrotron Radiation Facility (ESRF), 71, Avenue des Martyrs, Grenoble 38043, France; e Department of Mathematical Sciences, Durham University, Durham DH1 3LE, United Kingdom; University of St Andrews, United Kingdom

**Keywords:** anomalous dispersion, resonant scattering, diffraction spectroscopy, correction of the crystallographic model, synchrotron

## Abstract

Correcting for anomalous dispersion is part of any refinement of an X-ray dif­fraction crystal structure determination. The procedure takes the inelastic scattering in the diffraction experiment into account. This X-ray absorption effect is specific to each chemical compound and is particularly sensitive to radiation energies in the region of the absorption edges of the elements in the compound. Therefore, the widely used tabulated values for these corrections can only be approximations as they are based on calculations for isolated atoms. Features of the unique spatial and electronic environment that are directly related to the anomalous dispersion are ignored, although these can be observed spectroscopically. This significantly affects the fit between the crystallographic model and the measured intensities when the excitation wavelength in an X-ray diffraction experiment is close to an element’s absorption edge. Herein, we report on synchrotron multi-wavelength single-crystal X-ray diffraction, as well as X-ray absorption spectroscopy experiments which we performed on the mol­ecular compound Mo(CO)_6_ at energies around the molybdenum K edge. The dispersive (*f*′) and absorptive (*f*′′) terms of the anomalous dispersion can be refined as independent parameters in the full-matrix least-squares refinement. This procedure has been implemented as a new feature in the well-established *OLEX2* software suite. These refined parameters are in good agreement with the independently recorded X-ray absorption spectrum. The resulting crystallographic models show significant improvement compared to those employing tabulated values.

## Abstract 2

Refined anomalous dispersion values provide an easy and reliable way to correct for inelastic scattering effects of the electronic environment unique to each structure. Herein, we introduce the implementation of this refinement in *OLEX2* and discuss its usage and perspectives.

## 1. Introduction

With the increasing capabilities of X-ray diffraction equipment, the deficiencies of the conventional crystallographic model become more and more apparent. This model is based on the measured intensities of each Bragg reflection according to Equations (1) and (2), where the structure factor *F* is calculated by a sum over *N* atoms in the unit cell with the respective atomic form factor *f_n_* and the anisotropic displacement parameters **T**_*n*_.

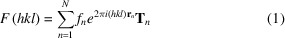




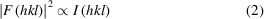




Resonant scattering, *i.e.* discrete electron transitions in the form of photoabsorption of incident radiation, is treated with the parameters *f*′ and *f*′′ – the real (dispersive) and imaginary (absorptive) part of the dispersion correction. Those are added to the atomic form factor *f_n_*_,0_ according to Equation (3).

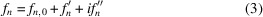




The impact of these parameters depends on the excitation energy, the type of atoms and their specific chemical bonding situation in the respective crystal structure. The original values for dispersion corrections commonly used so far are taken from tabulated libraries and based on calculations by Cromer (1965 ▸), Cromer & Liberman (1970 ▸) and Hönl (1933 ▸). They have been improved over several decades and are in good agreement with the experimental data of isolated atoms (Cromer & Mann, 1968 ▸). However, the tabulated literature values only consider the excitation energy and the atom type, completely ignoring the uniqueness of the atomic environment.

**Figure 1:**
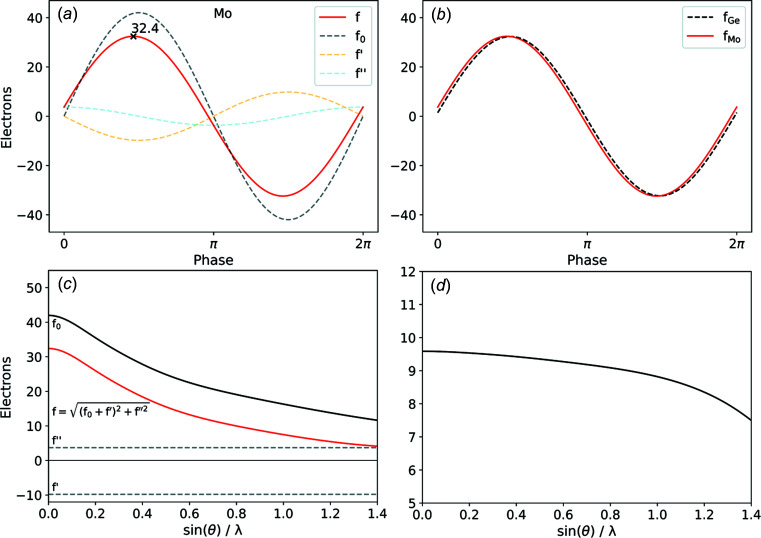
Dispersion corrections for the phase-dependent *f*(000) for (*a*) molybdenum and (*b*) comparison to germanium at 20 000 eV; (*c*) effect of *f*′ and *f*′′ on the atomic form factor *f*_0_; (*d*) resolution-dependent difference between *f*_0_ and *f*.

Fig. 1 ▸ shows an example of the effects of these correction terms for the atomic form factor of molybdenum at its K absorption edge at 20 000 eV. The real part of the correction *f*′ can be understood as a direct and the imaginary part *f*′′ as a phase-shifted contribution to the scattering power, which is constant across the entire range of resolution [see Fig. 1 ▸(*c*)]. As shown in Fig. 1 ▸(*a*), the amplitude of the effective atomic form factor *f* is significantly diminished by these dispersion corrections. At the K absorption edge of molybdenum (*Z* = 42), the resulting scattering power is reduced by as much as 9.6 electrons relative to a pure Thomson scattering model. At this energy, the corrected atomic form factor closely resembles the form factor of germanium [*Z* = 32; Fig. 1 ▸(*b*)]. In contrast to molybdenum, the germanium atomic form factor is only slightly affected by a dispersion correction at this energy. This effect even allows a wrongly assigned atom type to yield a similar or even better crystallographic model (Guss *et al.*, 1989 ▸).

Since the anomalous dispersion corrections are calculated for isolated non-inter­acting atoms, they differ significantly from those of atoms embedded in a particular chemical environment. Guss *et al.* (1989 ▸) have demonstrated this remarkable effect by recording an experimental X-ray absorption spectrum of a Cu^II^ metalloprotein; *f*′ was subsequently calculated based on the experimental *f*′′.

Equation (4) describes the proportionality relationship between *f*′′ and the linear absorption coefficient μ given by the frequency of the incident photon ω (Caticha-Ellis, 1981 ▸). The proportionality constants are the electron mass *m_e_*, the speed of light *c*, the number of scatterers *N* in the unit cell and the elementary electric charge *e*. Equation (5) describes how *f*′ is obtained as a contour integral around the K edge frequency ω_K_ (de Kronig, 1926 ▸; Kramers, 1927 ▸; Caticha-Ellis, 1981 ▸). These equations describe the link between an X-ray absorption spectrum and the crystallographic model of an X-ray diffraction experiment.

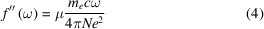




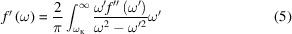




The frequency of an absorption edge is mostly determined by the given element. It has been shown that this frequency is further affected by the charge of the respective atom (Ankudinov, 1998 ▸). Spatzal *et al.* (2016 ▸) applied their spatially resolved anomalous dispersion (*SpReAD*) refinement to determine the individual oxidation states of the Fe atoms in nitro­genase from diffraction data (Einsle *et al.*, 2007 ▸). To the best of our knowledge, such an experimental determination of anomalous dispersion parameters was practically never carried out in small mol­ecule or solid-state crystallography.

The individual absorption spectrum exhibits additional features around the absorption edge which originate from electronic transitions into half-occupied and unoccupied orbitals, as well as into the continuum above the Fermi level (Hennig, 2007 ▸). Measuring these spectral features is the subject of the X-ray absorption near-edge structure (XANES) technique, and also of extended X-ray absorption fine structure (EXAFS) spectroscopy. The spectral fine structure at excitation energies above the absorption edge depends on the distances to neighbouring atoms and is therefore unique to each structure. None of these spectral features are considered in the tabulated dispersion values. Therefore, the application of these literature parameters is insufficient for X-ray crystallography, especially near absorption edges. Structures obtained with an incident photon energy near the absorption edge of a given atom show artefacts and result in poor crystallographic models (Dittrich *et al.*, 2015 ▸). This gave rise to a com­mon practice of avoiding single-crystal diffraction meas­urements near absorption edges.

## 2. Results and discussion

Since the exact energy of the absorption edge and the spectral features in the near vicinity to the edge are unique to the atom in its specific chemical environment, a simple approximation using calculations based on independent neutral atoms is always incorrect and leads to problems in the crystallographic model. Artefacts occur in the Fourier map and affect the overall scale factor. This effect was observed by Dittrich *et al.* (2015 ▸) who discuss anomalous dispersion effects in this context. In this case, even the wrong assignment of the atom type leads to apparently reliable structure models, which can even pass the common structure validation procedures (Spek, 2020 ▸). These authors also suggest difference electron-density plots relying on measurements above and below the absorption edge to visualize the effect of anomalous dispersion. In a later article, the same group reports on the opportunity to distinguish between neighbouring elements employing their different anomalous dispersion parameters even with laboratory sources in noncentrosymmetric space groups (Wandtke *et al.*, 2017 ▸). However, it should be pointed out that this also applies to centrosymmetric structures, as we show herein using the example of Mo(CO)_6_, which crystallizes in the centrosymmetric space group *Pnma*. Recent improvements in X-ray crystallography, such as new X-ray sources and detector types, but also the routine use of models based on nonspherical atomic form factors, increasingly reveal how inaccurate are the currently applied dispersion corrections.

To determine the effect of the dispersion correction on the quality of a crystallographic model, the example compound Mo(CO)_6_ was chosen. The K absorption edge of molybdenum occurs at exactly 20 000 eV, at which the Rossendorf beamline at the European Synchrotron Research Facility (ESRF) has excellent brilliance and suitable equipment to measure both the diffraction and the spectroscopic properties of single crystals and reference materials (Scheinost *et al.*, 2021 ▸).

A K edge absorption spectrum of a crystal of Mo(CO)_6_ was measured as a reference for the absorptive part of the scattering factors (*f*′′). The energy for this spectrum was calibrated against the first inflection point of a K edge spectrum of a molybdenum metal foil at 20 000 eV. The absorption edge of Mo(CO)_6_ was determined at 20 012 eV with a pre-edge at 20 001 eV and additional fine-structure derivative extrema at 20 018, 20 029 and 20 041 eV (see Fig. S1 in the supporting information). *f*′ was derived from the X-ray absorption spectrum according to Equation (5) using the program *kkcalc* (Watts, 2014 ▸).

Single-crystal X-ray diffraction experiments were performed at these energies, as well as at 19 900 and 20 100 eV as reference data well below and above the absorption edge. The diffraction data were corrected for absorption by em­ploy­ing the semi-empirical multi-scan routine, which is com­monly applied for redundant synchrotron data (Blessing, 1995 ▸). Since μ depends on *f*′′ according to Equation (4), no reasonable additional face-indexed absorption correction can be applied. The crystallographic software *OLEX2* was em­ploy­ed using *SHELXT* for the initial structure solution and *olex2.refine* as the refinement engine (Dolomanov *et al.*, 2009 ▸; Sheldrick, 2015 ▸; Bourhis *et al.*, 2015 ▸). Nonspherical atomic form factors were calculated with *NoSpherA2* (Kleemiss *et al.*, 2021 ▸), performing Hirshfeld-Atom-Refinement (HAR) em­ploy­ing a level of theory of DKH2-PBE0/x2c-TZVP within *ORCA* (Version 5.0; Capelli *et al.*, 2014 ▸; Jayatilaka & Dittrich, 2008 ▸; Neese *et al.*, 2020 ▸). HAR uses tailor-made form factors *f*_0_ computed from electron densities after a single-point wavefunction calculation for the current model, partitioned by Hirshfeld stockholder partitioning (Hirshfeld, 1976 ▸, 1977 ▸). The obtained atomic form factors are subsequently used in the refinement of the crystallographic model parameters, re­peat­ing form factor calculation and refinement until all parameters reach a convergence threshold.

To compare the effects of different dispersion parameters on the structure refinement, models were created using the values for molybdenum from the tables of Henke *et al.* (1993 ▸) and Sasaki (1989 ▸) for the respective energies. The latter closely resemble the values computed according to Brennan & Cowan (1992 ▸) (see Fig. S13 in the supporting information).

**Figure 2:**
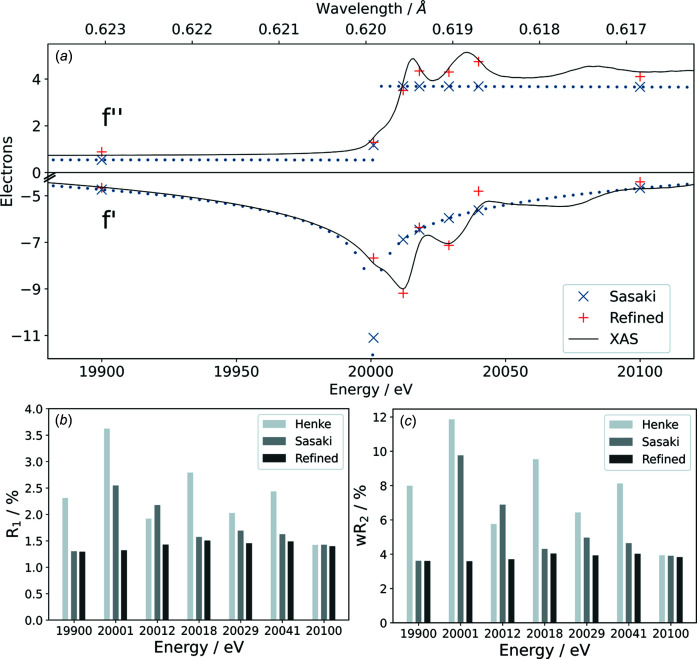
(*a*) X-ray absorption spectrum of Mo(CO)_6_ (top black line) and *f*′ calculated from it (bottom black line), Sasaki’s tabulated (x symbol blue) and refined values (+ symbol red) for *f*′′ and *f*′, and the resulting quality parameters (*b*) *R*_1_ and (*c*) *wR*_2_ of refined structure models.

The most commonly used source for dispersion correction parameters from the *Inter­national Tables for Crystallography* (Vol. C) are given for only a few energies used on standard laboratory diffractometers (Creagh & McAuley, 1992 ▸). The refinement of the dispersion correction for molybdenum was performed by the *olex2.refine* engine, which introduces *f*′ and *f*′′ as independent scalar parameters in the matrix least-squares refinement procedure (Bourhis *et al.*, 2015 ▸). The results of these calculations around the K edge of molybdenum and the obtained quality indicators are compared to those using the tabulated values in Fig. 2 ▸.

At 19 900 eV, Sasaki’s tabulated *f*′ and *f*′′ values agree well with both those obtained from the XAS data and the refined ones. The strongest deviation between the literature values and the refined dispersion parameters is observed at 20 001 eV. At this energy, the K edge is already exceeded in the tabulated values, while this is not yet the case for Mo(CO)_6_ according to the X-ray absorption spectrum. Above the absorption edge, the refined values follow the observed spectral fine structure. These features are specific to the individual crystal structure and cannot be captured by precalculated dispersion corrections. However, the values for *f*′ and *f*′′ can reliably be determined from the diffraction data. It is remarkable that here the smallest refined value of *f*′ is 3.75 electrons (e) above that of the tabulated values. The standard uncertainties of the dispersion values obtained by refinement is in the range 0.03–0.06 e (see Tables S1–S7 in the supporting information). The correlation between *f*′ and *f*′′ is low although they are related *via* Equation (5). This is due to the fact that the mutual dependence of *f*′ and *f*′′ includes the integral of a wide energy range, whereas the refinement is performed at a specific energy.

The large differences between *f*′ and *f*′′ are directly reflected in the structural models. The overall agreement factors vary between 1.30 < *R*_1_ < 3.62% and 3.62 < *wR*_2_ < 11.86% for the models with tabulated dispersion values. In contrast, the models obtained with refined dispersion corrections resulted in consistent agreement factors between 1.29 < *R*_1_ < 1.51% and 3.59 < *wR*_2_ < 4.03% (see Tables S1–S7 in the supporting information). Therefore, only the refinement of dispersion values leads to a robust model within the energy range investigated.

Furthermore, the deviations of the agreement factors are strongly correlated with the disagreement of the applied dispersion values to the X-ray absorption spectrum. This large deviation shows how drastically an incorrect dispersion correction can affect the crystallographic model if the incident energy is in the range of the absorption edge of an element involved.

**Figure 3:**
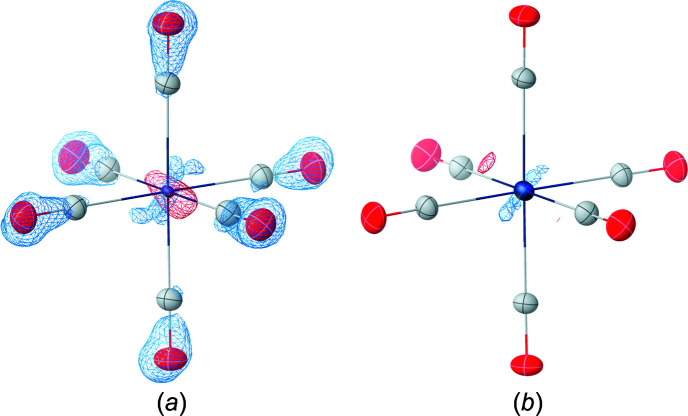
Negative (blue) and positive (red) residual electron density at an isosurface level of 0.33 e Å^−3^ of Mo(CO)_6_ at 20 001 eV for the HAR model using (*a*) the Sasaki tabulated and (*b*) refined dispersion values.

The effect on the crystallographic models can also be observed in the atomic displacement parameters (ADPs). Fig. 3 ▸ shows the models and difference Fourier maps at 20 001 eV using Sasaki tabulated dispersion values for molybdenum and refined ones, respectively. The unreasonably small displacement parameters of the metal atom in Fig. 3 ▸(*a*) are caused by the inadequate dispersion treatment according to the Sasaki tables, since the effective scattering power is overestimated. As a result, the residual electron-density map shows excessive electron density around the molybdenum position and electron depletion around the carbonyl ligands. This showcases the impact of one incorrect atomic form factor on the entire structure model. The biggest effect, however, is observed in the proximity of the metal centre.

A similar observation regarding the ADP size is known when an atom type is assigned to a position that contains more electrons than the modelled atom type provides. In the case of insufficient dispersion treatment, such a wrong assignment of an atom type leads to more reasonable anisotropic displacement parameters and refinement indicators. In contrast, the model with refined dispersion values in Fig. 3 ▸(*b*) shows none of these artefacts. The fractal dimension plots of the models (see Figs. S2 and S3 in the supporting information) according to Meindl & Henn (2008 ▸) also show a much better agreement. In addition, the precision of the C—O bond is best in all cases with the refined parameters (see Figs. S2 and S3). A more detailed discussion of the misassignment of atomic types in the Mo(CO)_6_ structure of this study and its implications for the atomic form factors is given in the supporting information.

## 3. Conclusion

We have shown that better treatment of the anomalous dis­persion correction is an important improvement to the conventional crystal structure determination. The inclusion of dispersion parameters *f*′ and *f*′′ in the least-squares refinement is simple and reliable, with low correlations and errors. The refined absorptive term *f*′′ follows the fine structure of the independently measured X-ray absorption of Mo(CO)_6_. The resulting crystallographic models are characterized by low *R*_1_ and *wR*_2_ values for the excitation energies chosen around the Mo K absorption edge. Conversely, the models using tabulated dispersion values are substanti­ally worse. The atomic displacement parameters, as well as the residual electron densities, are strongly affected by the incorrect dispersion treatment. The resulting effective form factor is incorrect by up to 3.7 e at 20 001 eV for *f*′ and 1.3 e for *f*′′ relative to the literature values.

We assume that the described method of dispersion refinement also influences crystal structures measured with laboratory diffractometers, especially when the available radiation falls near an absorption edge of a heavy element present in the compound. For instance, X-ray diffraction experiments with Cu *K*α radiation containing late first-row *d*-block metals or lanthanides will be affected. The latter elements even have three L absorption edges that occur in a broad energy range of about 2000 eV. In addition, laboratory diffractometers are operated with mixed *K*α_1,2_ radiation that differs by 20 eV for Cu, 105 eV for Mo and even 172 eV for Ag *K*α radiation, respectively. These energy differences would require two sets of dispersion parameters, which are individual for a crystal structure, to perform a proper correction for this effect. Since the *f*′′ parameter of the anomalous dispersion correction is directly related to the absorption coefficient μ, a resulting insufficient absorption correction can further worsen the results.

Therefore, a reconsideration of anomalous dispersion treat­ment will lead to a significant improvement of routine home source crystal structure determinations containing heavy elements.

## 4. Related literature

The following references are cited in the supporting information for this article: Dyadkin *et al.* (2016 ▸); Parsons *et al.* (2012 ▸); Ramseshan & Abrahams (1975 ▸).

## Supplementary Material

Crystal structure: contains datablock(s) MoC6O6_19900_solution_henke_nosphera2, MoC6O6_19900_solution_refined_nosphera2, MoC6O6_19900_solution_nosphera2, MoC6O6_20001_solution_henke_nosphera2, MoC6O6_20001_solution_nosphera2, MoC6O6_20001_solution_refined_nosphera2, MoC6O6_20012_solution_henke_nosphera2, MoC6O6_20012_solution_nosphera2, MoC6O6_20012_solution_refined_nosphera2, MoC6O6_20018_solution_henke_nosphera2, MoC6O6_20018_solution_nosphera2, MoC6O6_20018_solution_refined_nosphera2, MoC6O6_20029_solution_henke_nosphera2, MoC6O6_20029_solution_nosphera2, MoC6O6_20029_solution_refined_nosphera2, MoC6O6_20040_solution_henke_nosphera2, MoC6O6_20040_solution_nosphera2, MoC6O6_20040_solution_refined_nosphera2, MoC6O6_20100_solution_henke_nosphera2, MoC6O6_20100_solution_nosphera2, MoC6O6_20100_solution_refined_nosphera2, global. DOI: 10.1107/S2052252522006844/lt5050sup1.cif

laboratory diffractometer measurements. DOI: 10.1107/S2052252522006844/lt5050sup2.zip

leverage results. DOI: 10.1107/S2052252522006844/lt5050sup3.zip

Additional information. DOI: 10.1107/S2052252522006844/lt5050sup4.pdf

CCDC references: 2157649, 2157648, 2157647, 2157646, 2157645, 2157644, 2157643, 2157642, 2157641, 2157640, 2157639, 2157638, 2157637, 2157636, 2157635, 2157634, 2157633, 2157632, 2157630, 2157631, 2157629

## References

[bb1] Ankudinov, A. L., Ravel, B., Rehr, J. J. & Conradson, S. D. (1998). *Phys. Rev. B*, **58**, 7565–7576.

[bb2] Blessing, R. H. (1995). *Acta Cryst.* A**51**, 33–38. 10.1107/s0108767394005726 7702794

[bb3] Bourhis, L. J., Dolomanov, O. V., Gildea, R. J., Howard, J. A. K. & Puschmann, H. (2015). *Acta Cryst.* A**71**, 59–75. 10.1107/S2053273314022207 PMC4283469 25537389

[bb4] Brennan, S. & Cowan, P. L. (1992). *Rev. Sci. Instrum.* **63**, 850–853.

[bb5] Capelli, S. C., Bürgi, H.-B., Dittrich, B., Grabowsky, S. & Jayatilaka, D. (2014). *IUCrJ*, **1**, 361–379. 10.1107/S2052252514014845 PMC4174878 25295177

[bb6] Caticha-Ellis, S. (1981). In *Anomalous Dispersion of X-rays in Crystallography.* https://www.iucr.org/education/pamphlets/8/full-text.

[bb7] Creagh, D. C. & McAuley, W. (1992). *International Tables for Crystallography*, Vol. C, edited by A. J. C. Wilson, pp. 206–222. Dordrecht: Kluwer Academic Publishers.

[bb8] Cromer, D. T. (1965). *Acta Cryst.* **18**, 17–23.

[bb9] Cromer, D. T. & Liberman, D. (1970). *J. Chem. Phys.* **53**, 1891–1898.

[bb10] Cromer, D. T. & Mann, J. B. (1968). *Acta Cryst.* A**24**, 321–324.

[bb11] Dittrich, B., Wandtke, C. M., Meents, A., Pröpper, K., Mondal, K. C., Samuel, P. P., Amin SK, N., Singh, A. P., Roesky, H. W. & Sidhu, N. (2015). *ChemPhysChem*, **16**, 412–419. 10.1002/cphc.201402600 25393218

[bb12] Dolomanov, O. V., Bourhis, L. J., Gildea, R. J., Howard, J. A. K. & Puschmann, H. (2009). *J. Appl. Cryst.* **42**, 339–341.

[bb13] Dyadkin, V., Pattison, P., Dmitriev, V. & Chernyshov, D. (2016). *J. Synchrotron Rad.* **23**, 825–829. 10.1107/S1600577516002411 27140164

[bb14] Einsle, O., Andrade, S. L. A., Dobbek, H., Meyer, J. & Rees, D. C. (2007). *J. Am. Chem. Soc.* **129**, 2210–2211. 10.1021/ja067562o PMC2527600 17269774

[bb15] Guss, J. M., Merritt, E. A., Phizackerley, R. P., Hedman, B., Murata, M., Hodgson, K. O. & Freeman, H. C. (1988). *Science*, **241**, 806–811. 10.1126/science.3406739 3406739

[bb16] Henke, B. L., Gullikson, E. M. & Davis, J. C. (1993). *At. Data Nucl. Data Tables*, **54**, 181–342.

[bb17] Hennig, C. (2007). *Phys. Rev. B*, **75**, 035120.

[bb18] Hirshfeld, F. L. (1976). *Acta Cryst.* A**32**, 239–244.

[bb19] Hirshfeld, F. L. (1977). *Theor. Chim. Acta*, **44**, 129–138.

[bb20] Hönl, H. (1933). *Z. Phys.* **83**, 1–16.

[bb21] Jayatilaka, D. & Dittrich, B. (2008). *Acta Cryst.* A**64**, 383–393. 10.1107/S0108767308005709 18421128

[bb22] Kleemiss, F., Dolomanov, O. V., Bodensteiner, M., Peyerimhoff, N., Midgley, L., Bourhis, L. J., Genoni, A., Malaspina, L. A., Jayatilaka, D., Spencer, J. L., White, F., Grundkötter-Stock, B., Steinhauer, S., Lentz, D., Puschmann, H. & Grabowsky, S. (2021). *Chem. Sci.* **12**, 1675–1692. 10.1039/d0sc05526c PMC8179328 34163928

[bb23] Kramers, H. A. (1927). *Atti Congr. Int. Fis.* **2**, 545–557.

[bb24] Kronig, R. de L. (1926). *J. Opt. Soc. Am.* **12**, 547–706.

[bb25] Meindl, K. & Henn, J. (2008). *Acta Cryst.* A**64**, 404–418. 10.1107/S0108767308006879 18421130

[bb26] Neese, F., Wennmohs, F., Becker, U. & Riplinger, C. (2020). *J. Chem. Phys.* **152**, 224108. 10.1063/5.0004608 32534543

[bb27] Parsons, S., Wagner, T., Presly, O., Wood, P. A. & Cooper, R. I. (2012). *J. Appl. Cryst.* **45**, 417–429.

[bb28] Ramseshan, S. & Abrahams, S. C. (1975). Editors. *Anomalous Scattering*. Copenhagen: Munksgaard.

[bb29] Sasaki, S. (1989). KEK Report 88–14, pp. 1–136. Ibaraki-keri, Japan: National Laboratory for High Energy Physics.

[bb30] Scheinost, A. C., Claussner, J., Exner, J., Feig, M., Findeisen, S., Hennig, C., Kvashnina, K. O., Naudet, D., Prieur, D., Rossberg, A., Schmidt, M., Qiu, C., Colomp, P., Cohen, C., Dettona, E., Dyadkin, V. & Stumpf, T. (2021). *J. Synchrotron Rad.* **28**, 333–349. 10.1107/S1600577520014265 PMC7842221 33399586

[bb31] Sheldrick, G. M. (2015). *Acta Cryst.* A**71**, 3–8.

[bb32] Spatzal, T., Schlesier, J., Burger, E.-M., Sippel, D., Zhang, L., Andrade, S. L. A., Rees, D. C. & Einsle, O. (2016). *Nat. Commun.* **7**, 10902. 10.1038/ncomms10902 PMC4793075 26973151

[bb33] Spek, A. L. (2020). *Acta Cryst.* E**76**, 1–11. 10.1107/S2056989019016244 PMC6944088 31921444

[bb34] Wandtke, C. M., Weil, M., Simpson, J. & Dittrich, B. (2017). *Acta Cryst.* B**73**, 794–804. 10.1107/S2052520617010745 PMC5628397 28980983

[bb35] Watts, B. (2014). *Opt. Express*, **22**, 23628–23639. 10.1364/OE.22.023628 25321829

