# Supplementary material for: Refinement of anomalous dispersion correction parameters in single-crystal structure determinations
Source: IUCrJ. 2022 Jul 20;9(Pt 5):604–9. doi: 10.1107/S2052252522006844 (PMC9438505; doi:10.1107/S2052252522006844)
Supplement: Supplementary file 4 [file m-09-00604-sup4.pdf]

**IUCrJ**

**Volume 9 (2022)**

**Supporting information for article:**

# Refinement of anomalous dispersion correction parameters in single-crystal structure determinations

**Florian Meurer, Oleg V. Dolomanov, Christoph Hennig, Norbert Peyerimhoff, Florian Kleemiss, Horst Puschmann and Michael Bodensteiner**

# Supporting information

## S1. Experimental Procedures

A clear, colourless crystal of dimensions 0.1 × 0.1 × 0.1 $mm^3$ was mounted on a (SPECS) synchrotron diffractometer at the Rossendorf beamline BM20-CRG at the European Synchrotron Radiation Facility (ESRF) in Grenoble, France and irradiated with wavelengths corresponding to energies of 19,900 eV, 20,001 eV, 20,012 eV, 20,018 eV, 20,029 eV, 20,041 eV and 20,100 eV. The diffraction pattern was collected with a Dectris PILATUS3 X 2M detector module with 0.1° per image scan width. Compression and transformation of the data was performed using the *SNBL Tool Box* (Dyadkin *et al.*, 2016). Data processing, reduction and multi-scan absorption correction were performed with the program *CrysAlisPro* (Rigaku Oxford Diffraction, 2021). Further treatment and evaluation of the data was conducted in *Olex2* (Dolomanov *et al.*, 2009), where the structure was solved by the program *ShelXT* (Sheldrick, 2015) and refined using *olex2.refine* (Bourhis *et al.*, 2015). All refined values for dispersion correction were also obtained using the *olex2.refine* routine. To compute the non-spherical atomic form factors, the program *NoSpherA2* (Kleemiss *et al.*, 2021) was used and the wavefunction calculations were performed with *ORCA 5.0* (Neese *et al.*, 2020).

## S2. Refinement of *f´* and *f´´* in Olex2

The new feature is available in Olex2-1.5. The anomalous dispersion parameters can be included in the least-squares refinement of *olex2.refine* with commands "free" and "fix", which are already used in the software for the coordinates ("xyz"), occupancy ("occu") and displacement parameters ("uiso"). This macro is now extended by the new keyword "disp". The following examples illustrate the use:

`>> free disp` (refines *f´*/ *f´´* for all elements present in the structure)
`>> fix/free disp $Mo` (fixes/refines *f´*/ *f´´* for atom type molybdenum)
`>> fix/free disp Mo01` [or selection of an atom in the GUI] (fixes/refines *f´*/ *f´´* for atom Mo01)
`>> fix disp` (fixes *f´*/ *f´´* for all elements present in the structure)
`>> fix disp -c` (deletes all disp entries and *f´*/ *f´´* are no longer included in the refinement)
`>> same disp $Mo` (constrains all the dispersion values to be identical for all Mo atoms)

Tabulated values can be generated by

`>> gendisp -force -source=brennan` (other keywords “sasaki” and “henke”)

Compatibility with refinement software other than *olex2.refine* is kept with the refined *f´* and *f´´* parameters being added in REM lines of the ins/res file:

```
REM <dispersion
REM  <Mo01 "-4.9333 4.0371">
REM >
```

The refined values can be copied and used in the DISP instruction line of the .ins file to apply them in other software.

## S3. Mathematical clarification to Eq. 5

The integral

$$f'(\omega) = \frac{2}{\pi}\int_{\omega_K}^{\infty} \frac{\omega' f''(\omega')}{\omega^2 - \omega'^2}\mathrm{d}\omega' \qquad (5)$$

has to be understood as the Cauchy principal value, in the case $\omega \geq \omega_K$, defined as follows: (Ramseshan & Abrahams, 1975)

$$f'(\omega) = \frac{2}{\pi}\lim_{\epsilon\to\infty}\int_{\omega_K}^{\omega-\epsilon} \frac{\omega' f''(\omega')}{\omega^2 - \omega'^2}\mathrm{d}\omega' + \int_{\omega+\epsilon}^{\infty} \frac{\omega' f''(\omega')}{\omega^2 - \omega'^2}\mathrm{d}\omega' \qquad (S5)$$

## S4. Crystallographic data

**Table S1**: Crystallographic data for measurements of **1** at 19,900 eV and three sources for dispersion values

| Energy / eV | 19900 | 19900 | 19900 |
|---|---|---|---|
| Wavelength / Å | 0.62303 | 0.62303 | 0.62303 |
| Disp. Source | Henke | Sasaki | Refined |
| CCDC Nr. | 2157629 | 2157631 | 2157630 |
| Empirical formula | $MoC_6O_6$ | $MoC_6O_6$ | $MoC_6O_6$ |
| Formula weight / g·mol$^{-1}$ | 263.995 | 263.995 | 263.995 |
| Crystal size / mm$^{-3}$ | 0.1×0.1×0.1 | 0.1×0.1×0.1 | 0.1×0.1×0.1 |
| Temperature / K | 110 | 110 | 110 |
| *a* / Å | 11.74147(13) | 11.74147(13) | 11.74147(13) |
| *b* / Å | 11.22116(9) | 11.22116(9) | 11.22116(9) |
| *c* / Å | 6.35094(5) | 6.35094(5) | 6.35094(5) |
| α = β = γ / ° | 90 | 90 | 90 |
| V / Å$^{-3}$ | 836.755(13) | 836.755(13) | 836.755(13) |
| ρ / g·cm$^{-3}$ | 2.096 | 2.096 | 2.096 |
| μ / mm$^{-1}$ | 1.086 | 1.086 | 1.086 |
| θ-range / ° | 3.04 – 27.17 | 3.04 – 27.17 | 3.04 – 27.17 |
| Total reflections | 17051 | 17051 | 17051 |
| Unique reflections | 1407 | 1407 | 1407 |
| Completeness | 0.9987 | 0.9987 | 0.9987 |
| $R_1$, $wR_2$, $R_{int}$ / % | 2.31, 7.96, 3.38 | 1.30, 3.62, 3.38 | 1.29, 3.58, 3.38 |
| GooF | 1.022 | 1.045 | 1.038 |
| Largest diff. peak, hole / e·Å$^{-3}$ | 0.6672, -0.5214 | 0.6079, -0.4466 | 0.6021, -0.4559 |
| No. of restraints | 0 | 0 | 0 |
| No. of parameters | 68 | 68 | 70 |
| $I/\sigma(I)$ | 92.7 | 92.7 | 92.7 |
| Indices range | -16 ≥ h ≥ 16 | -16 ≥ h ≥ 16 | -16 ≥ h ≥ 16 |
| | -16 ≥ k ≥ 16 | -16 ≥ k ≥ 16 | -16 ≥ k ≥ 16 |
| | -9 ≥ l ≥ 9 | -9 ≥ l ≥ 9 | -9 ≥ l ≥ 9 |
| f’ / electrons | -4.7345 | -8.2545 | -4.65(5)* |
| f’’ / electrons | 0.5414 | 0.5562 | 0.88(6)* |
| C-O bond prec. / Å | 0.0024 | 0.0011 | 0.0010 |

* Correlation for f’ and f’’ in the least-squares refinement: 0.378

**Table S2**: Crystallographic data for measurements of **1** at 20,001 eV and three sources for dispersion values

| Energy / eV | 20001 | 20001 | 20001 |
|---|---|---|---|
| Wavelength / Å | 0.61988 | 0.61988 | 0.61988 |
| Disp. Source | Henke | Sasaki | Refined |
| CCDC Nr. | 2157632 | 2157633 | 2157634 |
| Empirical formula | $MoC_6O_6$ | $MoC_6O_6$ | $MoC_6O_6$ |
| Formula weight / g·mol$^{-1}$ | 263.995 | 263.995 | 263.995 |
| Crystal size / mm$^{-3}$ | 0.1×0.1×0.1 | 0.1×0.1×0.1 | 0.1×0.1×0.1 |
| Temperature / K | 110 | 110 | 110 |
| *a* / Å | 11.78656(17) | 11.78656(17) | 11.78656(17) |
| *b* / Å | 11.26190(12) | 11.26190(12) | 11.26190(12) |
| *c* / Å | 6.37330(7) | 6.37330(7) | 6.37330(7) |
| α = β = γ / ° | 90 | 90 | 90 |
| V / Å$^{-3}$ | 845.986(18) | 845.986(18) | 845.986(18) |
| ρ / g·cm$^{-3}$ | 2.073 | 2.073 | 2.073 |
| μ / mm$^{-1}$ | 6.156 | 6.156 | 6.156 |
| θ-range / ° | 3.01 – 27.09 | 3.01 – 27.09 | 3.01 – 27.09 |
| Total reflections | 17206 | 17206 | 17206 |
| Unique reflections | 1424 | 1424 | 1424 |
| Completeness | 1.0000 | 1.0000 | 1.0000 |
| $R_1$, $wR_2$, $R_{int}$ / % | 3.62, 11.79, 3.30 | 2.55, 9.69, 3.30 | 1.32, 3.55, 3.30 |
| GooF | 0.989 | 0.999 | 1.107 |
| Largest diff. peak, hole / e·Å$^{-3}$ | 1.3201, -0.5868 | 0.8075, -0.5318 | 0.4784, -0.3578 |
| No. of restraints | 0 | 0 | 0 |
| No. of parameters | 68 | 68 | 70 |
| $I/\sigma(I)$ | 91.2 | 91.2 | 91.2 |
| Indices range | -16 ≥ h ≥ 16 | -16 ≥ h ≥ 16 | -16 ≥ h ≥ 16 |
| | -16 ≥ k ≥ 16 | -16 ≥ k ≥ 16 | -16 ≥ k ≥ 16 |
| | -9 ≥ l ≥ 9 | -9 ≥ l ≥ 9 | -9 ≥ l ≥ 9 |
| f' / electrons | -11.1028 | -11.4284 | -7.68(3)* |
| f'' / electrons | 1.1680 | 3.7149 | 1.29(3)* |
| C-O bond prec. / Å | 0.0035 | 0.0018 | 0.0007 |

* Correlation for f' and f'' in the least-squares refinement: 0.322

**Table S3**: Crystallographic data for measurements of **1** at 20,012 eV and three sources for dispersion values

| Energy / eV | 20012 | 20012 | 20012 |
|---|---|---|---|
| Wavelength / Å | 0.61988 | 0.61988 | 0.61988 |
| Disp. Source | Henke | Sasaki | Refined |
| CCDC Nr. | 2157635 | 2157636 | 2157637 |
| Empirical formula | $MoC_6O_6$ | $MoC_6O_6$ | $MoC_6O_6$ |
| Formula weight / g·mol$^{-1}$ | 263.995 | 263.995 | 263.995 |
| Crystal size / mm$^{-3}$ | 0.1×0.1×0.1 | 0.1×0.1×0.1 | 0.1×0.1×0.1 |
| Temperature / K | 110 | 110 | 110 |
| *a* / Å | 11.7976(2) | 11.7976(2) | 11.7976(2) |
| *b* / Å | 11.2734(1) | 11.2734(1) | 11.2734(1) |
| *c* / Å | 6.3799(1) | 6.3799(1) | 6.3799(1) |
| α = β = γ / ° | 90 | 90 | 90 |
| V / Å$^{-3}$ | 848.52(2) | 848.52(2) | 848.52(2) |
| ρ / g·cm$^{-3}$ | 2.067 | 2.067 | 2.067 |
| μ / mm$^{-1}$ | 6.123 | 6.123 | 6.123 |
| θ-range / ° | 3.01 – 27.04 | 3.01 – 27.04 | 3.01 – 27.04 |
| Total reflections | 17325 | 17325 | 17325 |
| Unique reflections | 1428 | 1428 | 1428 |
| Completeness | 1.0000 | 1.0000 | 1.0000 |
| $R_1$, $wR_2$, $R_{int}$ / % | 1.92, 5.72, 3.54 | 2.18, 6.82, 3.54 | 1.43, 3.70, 3.54 |
| GooF | 1.012 | 1.008 | 1.063 |
| Largest diff. peak, hole / e·Å$^{-3}$ | 0.5191, -0.3221 | 0.5454, -0.6531 | 0.4889, -0.3267 |
| No. of restraints | 0 | 0 | 0 |
| No. of parameters | 68 | 68 | 70 |
| $I/\sigma(I)$ | 83.0 | 83.0 | 83.0 |
| Indices range | -16 ≥ h ≥ 16 | -16 ≥ h ≥ 16 | -16 ≥ h ≥ 16 |
| | -16 ≥ k ≥ 16 | -16 ≥ k ≥ 16 | -16 ≥ k ≥ 16 |
| | -9 ≥ l ≥ 9 | -9 ≥ l ≥ 9 | -9 ≥ l ≥ 9 |
| f' / electrons | -6.8798 | -10.5678 | -9.19(4)* |
| f'' / electrons | 3.6915 | 3.7117 | 3.51(3)* |
| C-O bond prec. / Å | 0.0015 | 0.0015 | 0.0008 |

* Correlation for f' and f'' in the least-squares refinement: 0.318

**Table S4**: Crystallographic data for measurements of **1** at 20,018 eV and three sources for dispersion values

| Energy / eV | 20018 | 20018 | 20018 |
|---|---|---|---|
| Wavelength / Å | 0.61936 | 0.61936 | 0.61936 |
| Disp. Source | Henke | Sasaki | Refined |
| CCDC Nr. | 2157638 | 2157639 | 2157640 |
| Empirical formula | $MoC_6O_6$ | $MoC_6O_6$ | $MoC_6O_6$ |
| Formula weight / g·mol$^{-1}$ | 263.995 | 263.995 | 263.995 |
| Crystal size / mm$^{-3}$ | 0.1×0.1×0.1 | 0.1×0.1×0.1 | 0.1×0.1×0.1 |
| Temperature / K | 110 | 110 | 110 |
| *a* / Å | 11.80112(14) | 11.80112(14) | 11.80112(14) |
| *b* / Å | 11.27663(9) | 11.27663(9) | 11.27663(9) |
| *c* / Å | 6.38185(5) | 6.38185(5) | 6.38185(5) |
| α = β = γ / ° | 90 | 90 | 90 |
| V / Å$^{-3}$ | 849.277(14) | 849.277(14) | 849.277(14) |
| ρ / g·cm$^{-3}$ | 2.065 | 2.065 | 2.065 |
| μ / mm$^{-1}$ | 6.118 | 6.118 | 6.118 |
| θ-range / ° | 3.01 -27.03 | 3.01 -27.03 | 3.01 -27.03 |
| Total reflections | 17340 | 17340 | 17340 |
| Unique reflections | 1430 | 1430 | 1430 |
| Completeness | 1.0000 | 1.0000 | 1.0000 |
| $R_1$, $wR_2$, $R_{int}$ / % | 2.79, 9.44, 3.67 | 1.57, 4.26, 3.67 | 1.51, 3.99, 3.67 |
| GooF | 1.012 | 1.043 | 1.055 |
| Largest diff. peak, hole / e·Å$^{-3}$ | 1.0324, -0.5775 | 0.5978, -0.4321 | 0.5909, -0.4326 |
| No. of restraints | 0 | 0 | 0 |
| No. of parameters | 68 | 68 | 70 |
| $I/\sigma(I)$ | 83.8 | 83.8 | 83.8 |
| Indices range | -16 ≥ h ≥ 16 | -16 ≥ h ≥ 16 | -16 ≥ h ≥ 16 |
| | -16 ≥ k ≥ 16 | -16 ≥ k ≥ 16 | -16 ≥ k ≥ 16 |
| | -9 ≥ l ≥ 9 | -9 ≥ l ≥ 9 | -9 ≥ l ≥ 9 |
| f' / electrons | -6.4606 | -10.118 | -6.36(6)* |
| f'' / electrons | 3.6895 | 3.7100 | 4.34(4)* |
| C-O bond prec. / Å | 0.0018 | 0.0012 | 0.0011 |

* Correlation for f' and f'' in the least-squares refinement: 0.258

**Table S5**: Crystallographic data for measurements of **1** at 20,029 eV and three sources for dispersion values

| Energy / eV | 20029 | 20029 | 20029 |
|---|---|---|---|
| Wavelength / Å | 0.61902 | 0.61902 | 0.61902 |
| Disp. Source | Henke | Sasaki | Refined |
| CCDC Nr. | 2157641 | 2157642 | 2157643 |
| Empirical formula | $MoC_6O_6$ | $MoC_6O_6$ | $MoC_6O_6$ |
| Formula weight / g·mol$^{-1}$ | 263.995 | 263.995 | 263.995 |
| Crystal size / mm$^{-3}$ | 0.1×0.1×0.1 | 0.1×0.1×0.1 | 0.1×0.1×0.1 |
| Temperature / K | 110 | 110 | 110 |
| *a* / Å | 11.80973(13) | 11.80973(13) | 11.80973(13) |
| *b* / Å | 11.28599(8) | 11.28599(8) | 11.28599(8) |
| *c* / Å | 6.38709(5) | 6.38709(5) | 6.38709(5) |
| α = β = γ / ° | 90 | 90 | 90 |
| V / Å$^{-3}$ | 851.299(13) | 851.299(13) | 851.299(13) |
| ρ / g·cm$^{-3}$ | 2.060 | 2.060 | 2.060 |
| μ / mm$^{-1}$ | 6.088 | 6.088 | 6.088 |
| θ-range / ° | 3.00 – 26.99 | 3.00 – 26.99 | 3.00 – 26.99 |
| Total reflections | 17449 | 17449 | 17449 |
| Unique reflections | 1430 | 1430 | 1430 |
| Completeness | 0.9988 | 0.9988 | 0.9988 |
| $R_1$, $wR_2$, $R_{int}$ / % | 2.03, 6.38, 3.70 | 1.69, 4.93, 3.70 | 1.45, 3.89, 3.70 |
| GooF | 1.063 | 1.026 | 1.083 |
| Largest diff. peak, hole / e·Å$^{-3}$ | 0.5900, -0.4143 | 0.6135, -0.4087 | 0.5854, -0.3622 |
| No. of restraints | 0 | 0 | 0 |
| No. of parameters | 68 | 68 | 70 |
| $I/\sigma(I)$ | 83.3 | 83.3 | 83.3 |
| Indices range | -16 ≥ h ≥ 16 | -16 ≥ h ≥ 16 | -16 ≥ h ≥ 16 |
| | -16 ≥ k ≥ 16 | -16 ≥ k ≥ 16 | -16 ≥ k ≥ 16 |
| | -9 ≥ l ≥ 9 | -9 ≥ l ≥ 9 | -9 ≥ l ≥ 9 |
| f’ / electrons | -5.9592 | -9.2498 | -7.13(5)* |
| f’’ / electrons | 3.6857 | 3.7068 | 4.30(3)* |
| C-O bond prec. / Å | 0.0012 | 0.0015 | 0.0010 |

* Correlation for f’ and f’’ in the least-squares refinement: 0.290

**Table S6**: Crystallographic data for measurements of **1** at 20,041 eV and three sources for dispersion values

| Energy / eV | 20041 | 20041 | 20041 |
|---|---|---|---|
| Wavelength / Å | 0.61868 | 0.61868 | 0.61868 |
| Disp. Source | Henke | Sasaki | Refined |
| CCDC Nr. | 2157644 | 2157645 | 2157646 |
| Empirical formula | $MoC_6O_6$ | $MoC_6O_6$ | $MoC_6O_6$ |
| Formula weight / g·mol$^{-1}$ | 263.995 | 263.995 | 263.995 |
| Crystal size / mm$^{-3}$ | 0.1×0.1×0.1 | 0.1×0.1×0.1 | 0.1×0.1×0.1 |
| Temperature / K | 110 | 110 | 110 |
| *a* / Å | 11.8215(12) | 11.8215(12) | 11.8215(12) |
| *b* / Å | 11.29740(8) | 11.29740(8) | 11.29740(8) |
| *c* / Å | 6.39402(5) | 6.39402(5) | 6.39402(5) |
| α = β = γ / ° | 90 | 90 | 90 |
| V / Å$^{-3}$ | 853.918(12) | 853.918(12) | 853.918(12) |
| ρ / g·cm$^{-3}$ | 2.053 | 2.053 | 2.053 |
| μ / mm$^{-1}$ | 6.041 | 6.041 | 6.041 |
| θ-range / ° | 3.00 – 26.95 | 3.00 – 26.95 | 3.00 – 26.95 |
| Total reflections | 17479 | 17479 | 17479 |
| Unique reflections | 1433 | 1433 | 1433 |
| Completeness | 0.9988 | 0.9988 | 0.9988 |
| $R_1$, $wR_2$, $R_{int}$ / % | 2.44, 8.03, 3.81 | 1.63, 4.60, 3.81 | 1.49, 3.98, 3.81 |
| GooF | 1.025 | 1.043 | 1.061 |
| Largest diff. peak, hole / e·Å$^{-3}$ | 0.7932, -0.582 | 0.7771, -0.3572 | 0.7748, -0.4460 |
| No. of restraints | 0 | 0 | 0 |
| No. of parameters | 68 | 68 | 70 |
| $I/\sigma(I)$ | 80.4 | 80.4 | 80.4 |
| Indices range | -16 ≥ h ≥ 16 | -16 ≥ h ≥ 16 | -16 ≥ h ≥ 16 |
| | -16 ≥ k ≥ 16 | -16 ≥ k ≥ 16 | -16 ≥ k ≥ 16 |
| | -9 ≥ l ≥ 9 | -9 ≥ l ≥ 9 | -9 ≥ l ≥ 9 |
| f' / electrons | -5.6242 | -8.3868 | -4.81(6)* |
| f'' / electrons | 3.6819 | 3.7036 | 4.74(4)* |
| C-O bond prec. / Å | 0.0018 | 0.0015 | 0.0012 |

* Correlation for f' and f'' in the least-squares refinement: 0.188

**Table S7**: Crystallographic data for measurements of **1** at 20,100 eV and three sources for dispersion values

| Energy / eV | 20100 | 20100 | 20100 |
|---|---|---|---|
| Wavelength / Å | 0.61683 | 0.61683 | 0.61683 |
| Disp. Source | Henke | Sasaki | Refined |
| CCDC Nr. | 2157647 | 2157648 | 2157649 |
| Empirical formula | $MoC_6O_6$ | $MoC_6O_6$ | $MoC_6O_6$ |
| Formula weight / g·mol$^{-1}$ | 263.995 | 263.995 | 263.995 |
| Crystal size / mm$^3$ | 0.1×0.1×0.1 | 0.1×0.1×0.1 | 0.1×0.1×0.1 |
| Temperature / K | 110 | 110 | 110 |
| *a* / Å | 11.86220(11) | 11.86220(11) | 11.86220(11) |
| *b* / Å | 11.33684(8) | 11.33684(8) | 11.33684(8) |
| *c* / Å | 6.41647(4) | 6.41647(4) | 6.41647(4) |
| α = β = γ / ° | 90 | 90 | 90 |
| V / Å$^{-3}$ | 862.886(12) | 862.886(12) | 862.886(12) |
| ρ / g·cm$^{-3}$ | 2.032 | 2.032 | 2.032 |
| μ / mm$^{-1}$ | 5.899 | 5.899 | 5.899 |
| θ-range / ° | 2.98 – 26.87 | 2.98 – 26.87 | 2.98 – 26.87 |
| Total reflections | 17604 | 17604 | 17604 |
| Unique reflections | 1446 | 1446 | 1446 |
| Completeness | 0.9976 | 0.9976 | 0.9976 |
| $R_1$, $wR_2$, $R_{int}$ / % | 1.43, 3.90, 3.71 | 1.43, 3.91, 3.71 | 1.40, 3.83, 3.71 |
| GooF | 1.060 | 1.060 | 1.069 |
| Largest diff. peak, hole / e·Å$^{-3}$ | 0.6906, -0.3543 | 0.6888, -0,3533 | 0.6793, -0.3730 |
| No. of restraints | 0 | 0 | 0 |
| No. of parameters | 68 | 68 | 70 |
| $I/\sigma(I)$ | 85.3 | 85.3 | 85.3 |
| Indices range | -16 ≥ h ≥ 16 | -16 ≥ h ≥ 16 | -16 ≥ h ≥ 16 |
| | -16 ≥ k ≥ 16 | -16 ≥ k ≥ 16 | -16 ≥ k ≥ 16 |
| | -9 ≥ l ≥ 9 | -9 ≥ l ≥ 9 | -9 ≥ l ≥ 9 |
| f’ / electrons | -4.6704 | -4.6374 | -4.40(6)* |
| f’’ / electrons | 3.6613 | 3.6862 | 4.10(4)* |
| C-O bond prec. / Å | 0.0011 | 0.0012 | 0.0011 |

* Correlation for f’ and f’’ in the least-squares refinement: 0.119

### S5. Spectra of Mo-metal and $Mo(CO)_6$

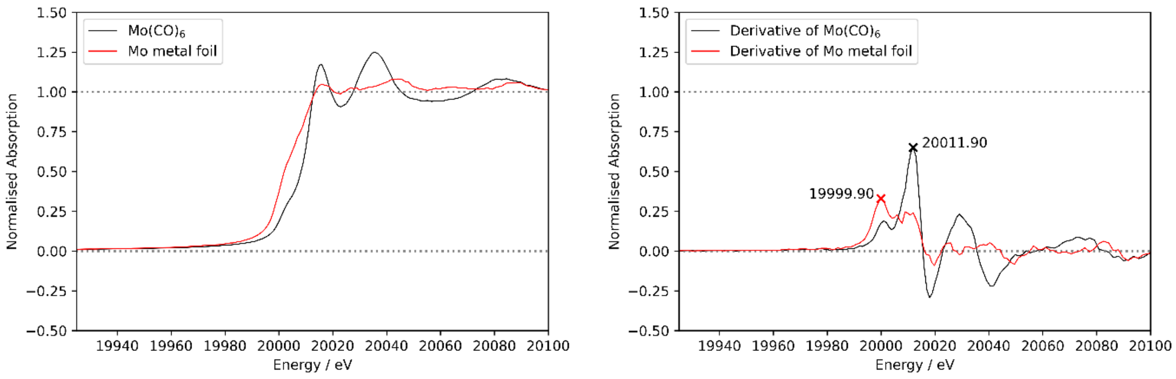


**A** **B**

**Fig. S1**: Comparison of the obtained X-ray absorption spectra (**A**) of both Mo metal foil and $Mo(CO)_6$ and the respective derivatives (**B**) used to determine the K absorption edge.

### S6. Fractal dimension / normal probability plots

In both models based on the tabulated dispersion values, a broadened shoulder towards a positive residual density is visible in the fractal dimension plots. (Meindl & Henn, 2008) The model based on the table of Henke *et al.* (1993) shows a more pronounced shoulder than the model based on Sasaki's (1989). In contrast, the plot obtained employing the refined dispersion correction shows only a slight deformation of the two underlying normal distributions. This illustrates the drastic effect of the shift of the absorption edge of molybdenum in $Mo(CO)_6$ in contrast to elemental metal.

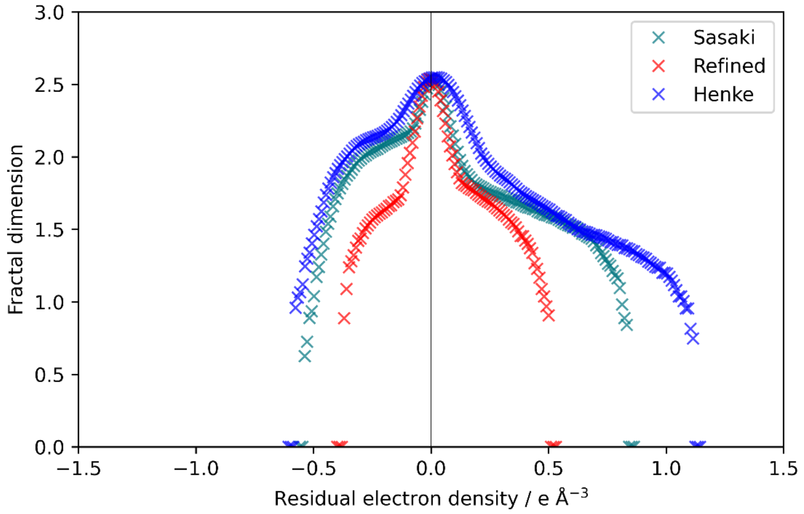


**Fig. S2**: Fractal dimension distributions according to Meindl & Henn (2008) of **1** at 20,001 eV of the crystallographic model using tabulated dispersion values according to Henke (blue), Sasaki (green) or refined ones (red).

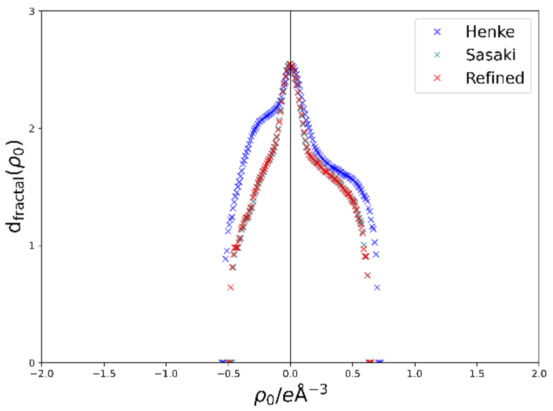


19,900 eV

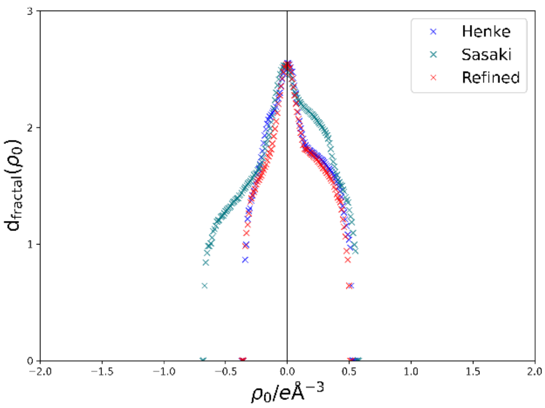


20,012 eV

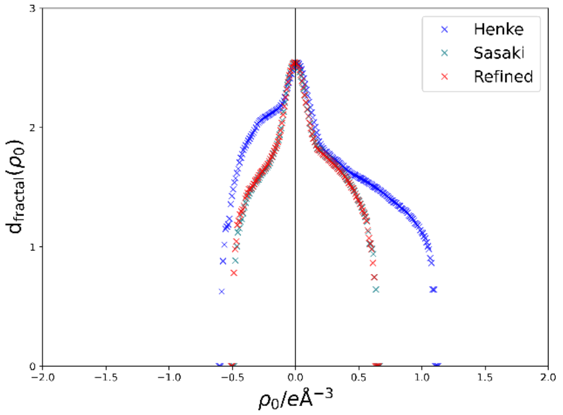


20,018 eV

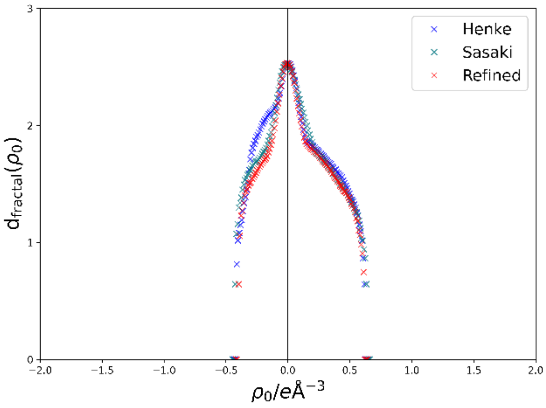


20,029 eV

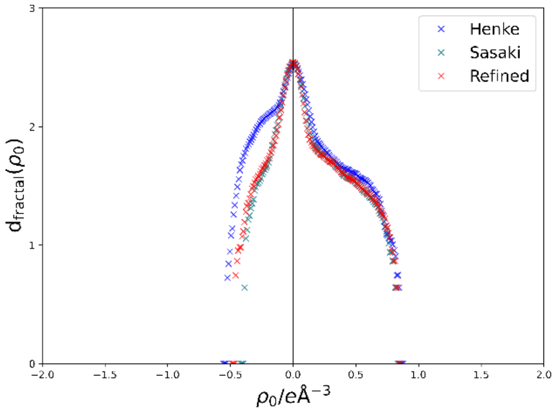


20,040 eV

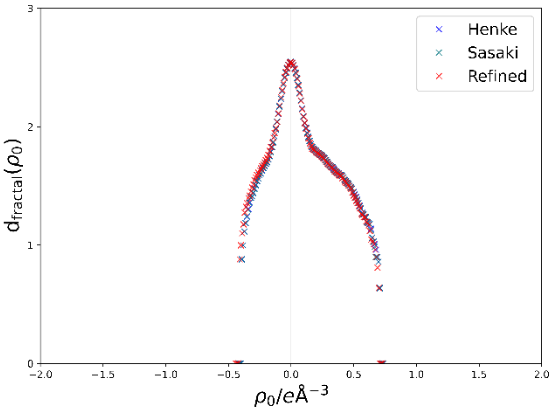


20,100 eV

**Fig. S3**: Fractal dimension plots of **1** at different energies.

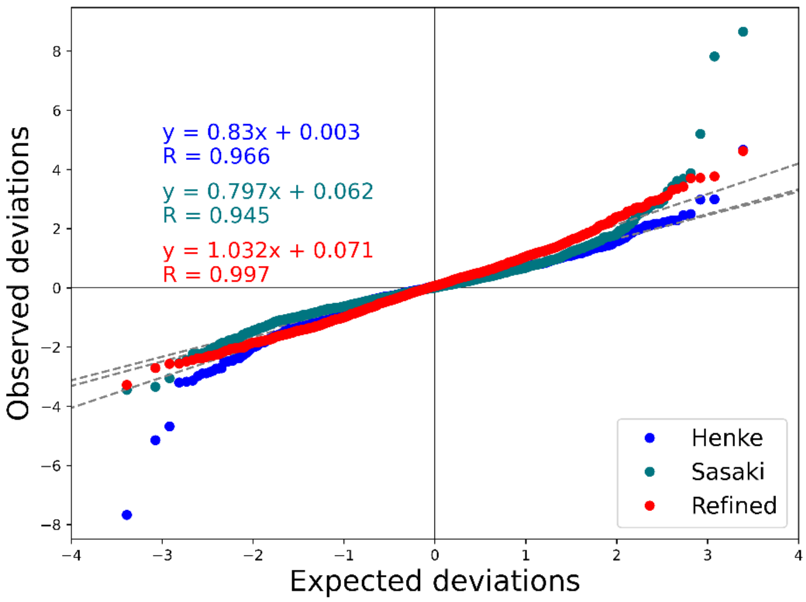


**Fig. S4**: Normal probability plots of **1** at 20,012 eV of the crystallographic model using tabulated dispersion values according to Henke (blue), Sasaki (green) or refined ones (red).

The normal probability plot (Fig. S4) shows the smallest deviations for the model based on refined dispersion parameters. Both models based on tabulated values show strong deviations from the expected distribution. At energies above the absorption edge, the model based on refined dispersion parameters shows increasing deviations (Fig. S5).

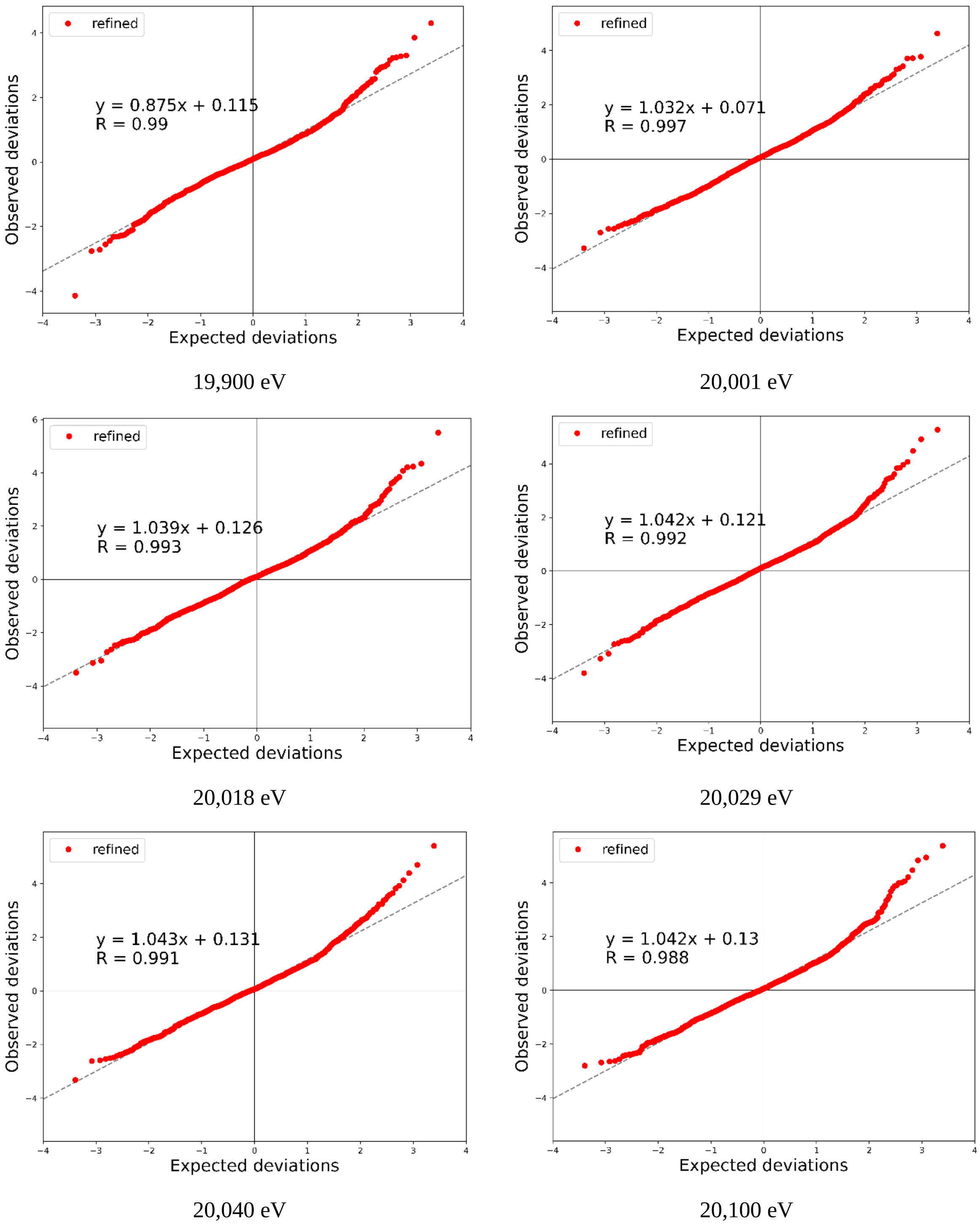


**Fig. S5**: Normal probability plots for **1** with refined anomalous dispersion parameters at different energies.

## S7. Additional residual density plots

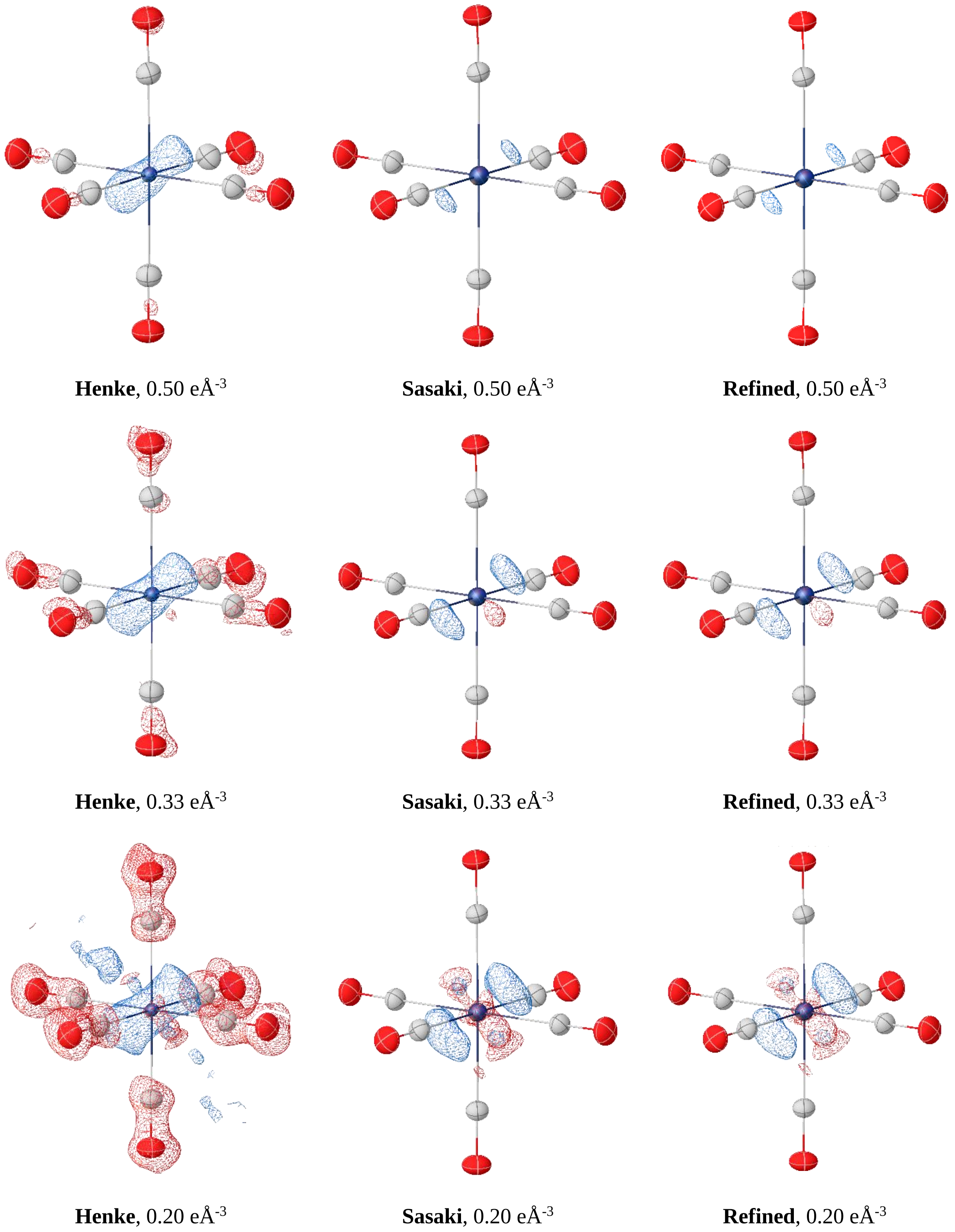


**Fig. S6**: Residual electron density plots of **1** measured at 19,900 eV for crystallographic models based on the Sasaki, Henke or refined anomalous dispersion parameters.

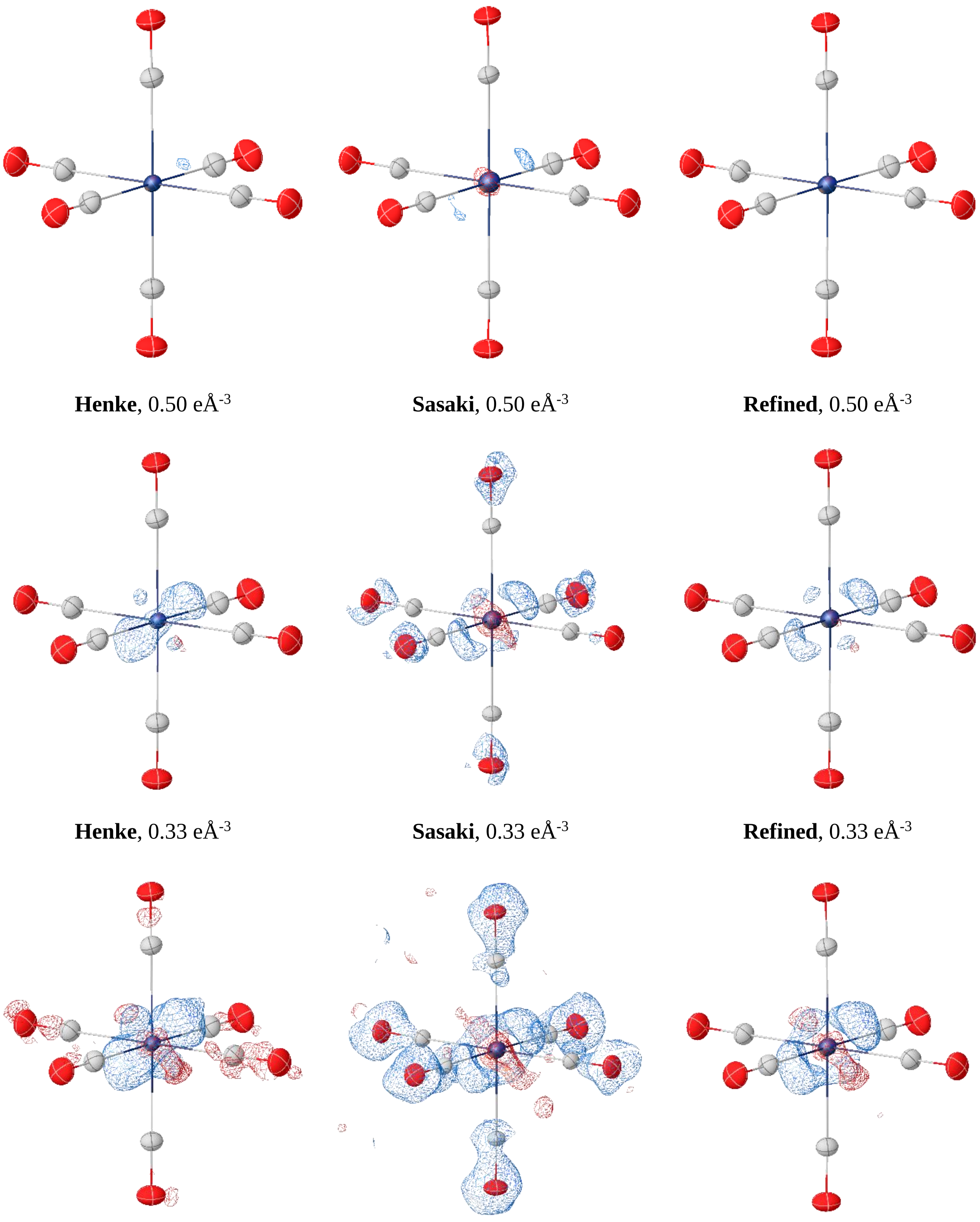

**Henke**, 0.50 eÅ$^{-3}$ **Sasaki**, 0.50 eÅ$^{-3}$ **Refined**, 0.50 eÅ$^{-3}$

**Henke**, 0.33 eÅ$^{-3}$ **Sasaki**, 0.33 eÅ$^{-3}$ **Refined**, 0.33 eÅ$^{-3}$

**Henke**, 0.20 eÅ$^{-3}$ **Sasaki**, 0.20 eÅ$^{-3}$ **Refined**, 0.20 eÅ$^{-3}$

**Fig. S7**: Residual electron density plots of **1** measured at 20,012 eV for crystallographic models based on the Sasaki, Henke or refined anomalous dispersion parameters.

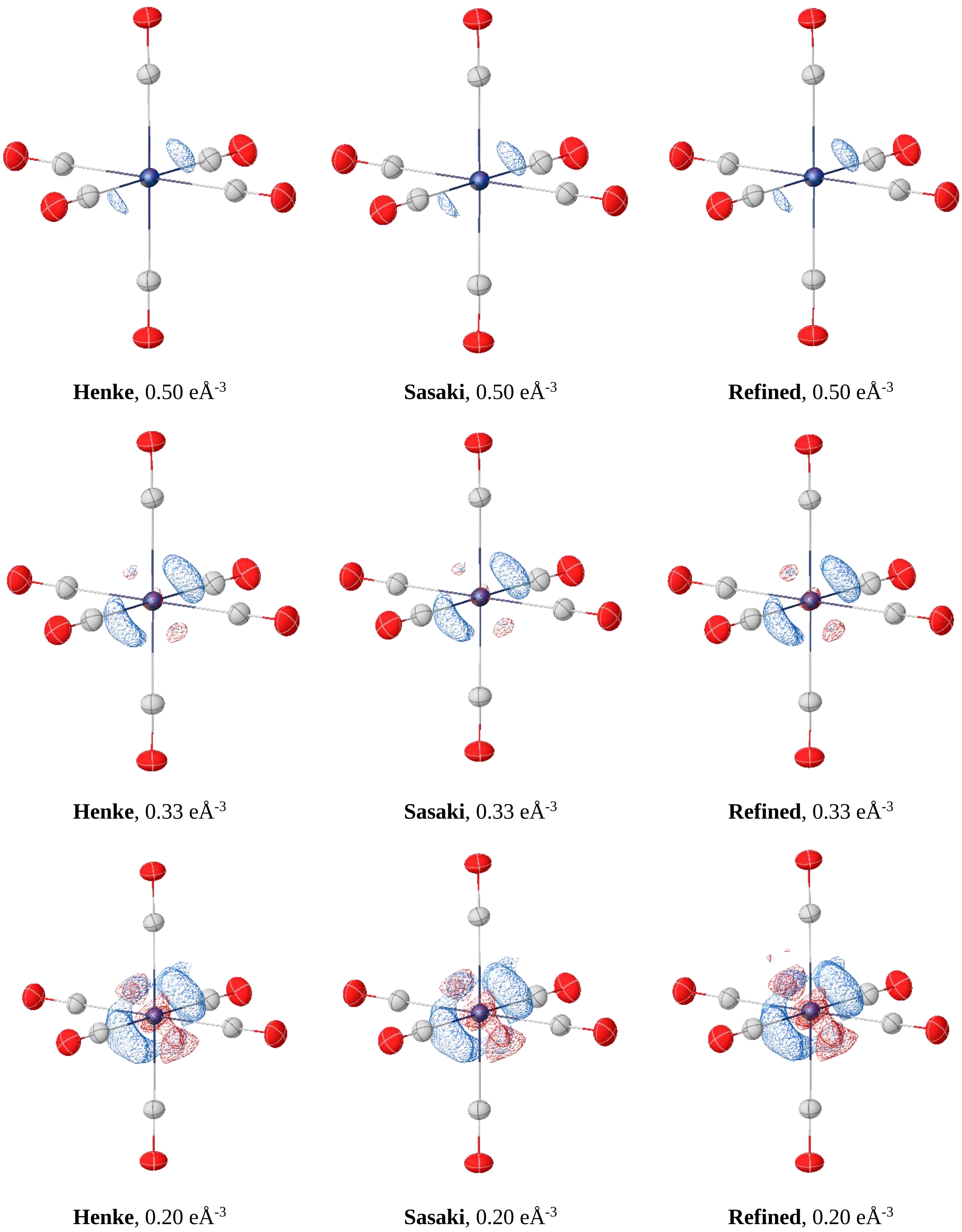


**Fig. S8**: Residual electron density plots of **1** measured at 20,100 eV for crystallographic models based on the Sasaki, Henke or refined anomalous dispersion parameters.

### S8. Intentionally wrong atom assignment

To demonstrate the strong effect of insufficient correction for anomalous dispersion, the molybdenum atom in $Mo(CO)_6$ was replaced with several other elements in the independent atom model and then refined against the data obtained at 20,001 eV, i.e. at the absorption edge of elemental molybdenum, but not yet that of $Mo(CO)_6$.

**Tab. S8A**: Quality indicators for independent atom models of $E(CO)_6$ (E = Element).

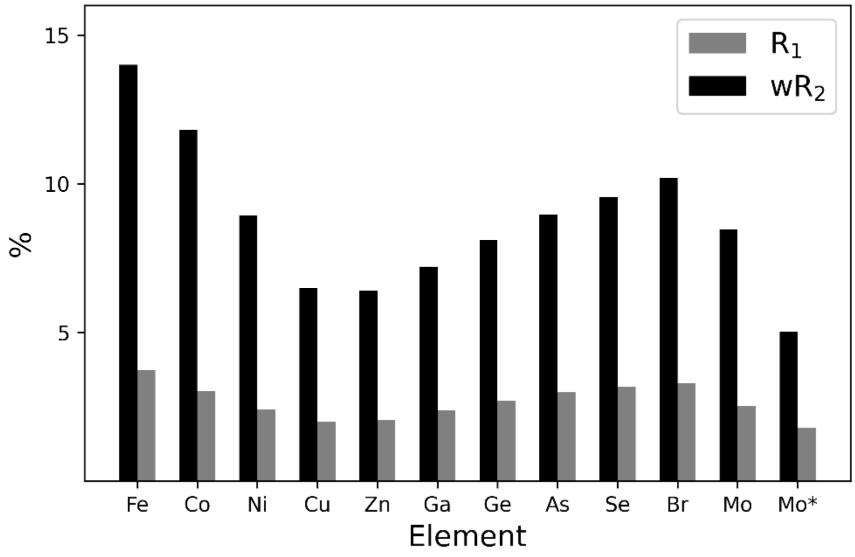


| Element | $R_1$, $wR_2$ / % |
|---|---|
| Fe | 3.74, 14.0 |
| Co | 3.03, 11.8 |
| Ni | 2.42, 8.93 |
| Cu | 2.00, 6.50 |
| Zn | 2.05, 6.40 |
| Ga | 2.38, 7.21 |
| Ge | 2.72, 8.10 |
| As | 2.99, 8.96 |
| Se | 3.17, 9.56 |
| Br | 3.29, 10.2 |
| Mo | 2.54, 8.46 |
| Mo* | 1.80, 5.03 |

**A** **B**

**Fig. S9**: $R_1$ and $wR_2$ of the crystallographic models of $E(CO)_6$ with the respective fourth-row element and Mo based on the Sasaki (1989) table and Mo* with refined dispersion parameters.

Replacing the molybdenum ($Z$ = 42) atom with elements $26 \leq Z \leq 35$ leads to reasonable quality indicators. Four wrong elements (Ni, Cu, Zn, Ga) provide lower $R_1$ and $wR_2$ values than the molybdenum model based on the Sasaki (1989) table for dispersion correction. Refining the dispersion parameters of molybdenum yields the best results.

To find out why the model with copper performs the best, the atomic form factors of these atoms were compared. Each of them consists of a sum of four Gaussian functions with different tabulated fitting coefficients $a$, $b$ and an additional offset parameter $c$ according to the following equation with $Q$ being the scattering vector:

$$f(Q) = \sum_{i=1}^{4} a_i \exp\left(-b_i \left(\frac{Q}{4\pi}\right)^2\right) + c \quad \text{with} \quad Q = 2 \cdot k \cdot \sin\theta \quad \text{and} \quad k = \frac{2\pi}{\lambda}$$

Dispersion correction subsequently reduces the atomic form factor. Comparing the form factors of the other elements with that of molybdenum, they are far apart when the dispersion correction is not considered, but very close when it is.

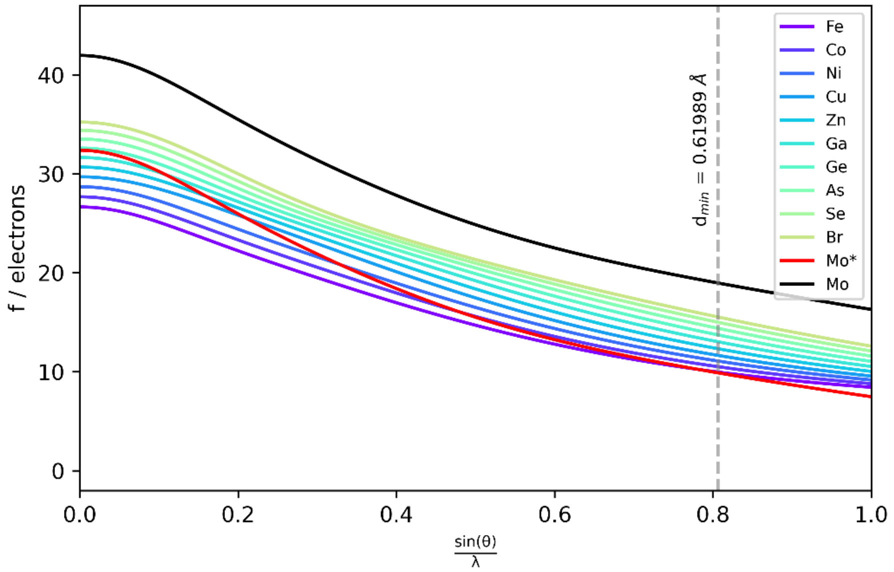


**Fig. S10**: Sasaki dispersion corrected atomic form factors for the elements Fe to Br and Mo as well as molybdenum with refined dispersion (Mo*) at 20,001 eV.

From Fig. S10 it is not directly obvious why copper and zinc fit best, but the difference between their atomic form factor and the corrected one of molybdenum is least.

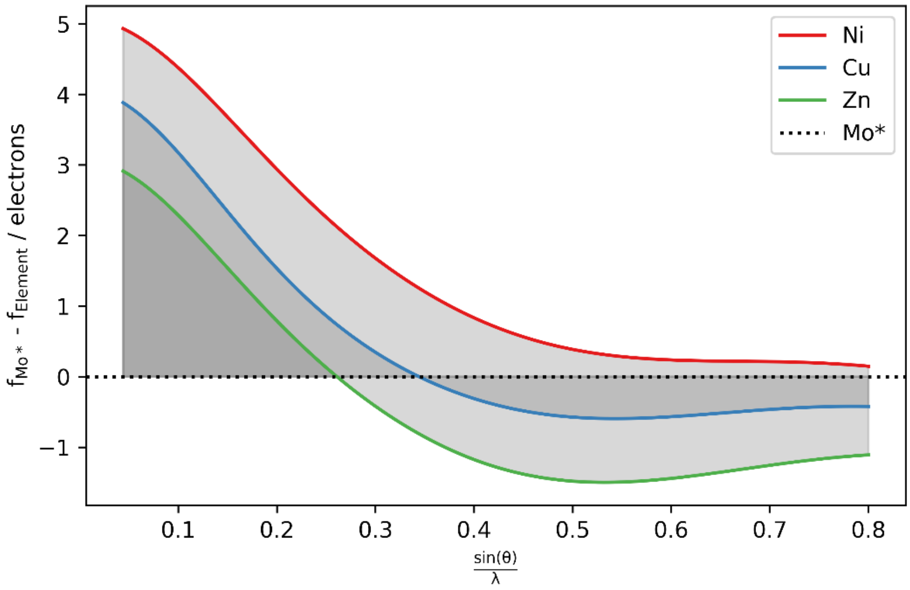


**Fig. S11**: Deviations of the Sasaki corrected form factors from the refined dispersion corrected ones of molybdenum (Mo*) within the experimental resolution range: The integrals of the curves are: Ni 1.07 $e^-$, Cu 0.424 $e^-$ and Zn -0.402 $e^-$.

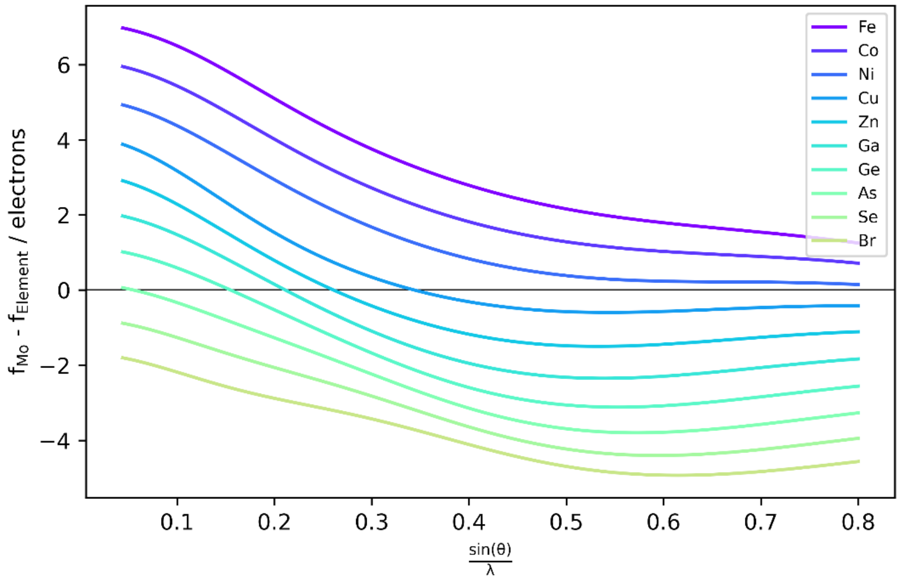


**Fig. S12**: Difference between the Sasaki corrected atomic form factors of selected elements relative to the refined dispersion corrected atomic form factor of molybdenum (Mo*) within the experimental resolution range.

If the anomalous dispersion parameters of the wrong atom models are refined, non-physical values for *f´* are obtained which compensate the wrong atomic form factor. However, it is remarkable, that *f´´* remains almost unchanged for all elements.

**Tab. S8B:** Wrong atom type models with their refined anomalous dispersion and quality parameters

| Element | refined *f´*, *f´´* / electrons | Sasaki (1989) *f´*, *f´´* / electrons | $R_1$, $wR_2$ / % |
|---|---|---|---|
| Fe | 5.22, 1.26 | 0.3201, 0.6612 | 1.90, 5.88 |
| Co | 4.04, 1.24 | 0.3322, 0.7625 | 1.93, 5.88 |
| Ni | 2.85, 1.23 | 0.3370, 0.8738 | 1.96, 6.01 |
| Cu | 1.39, 1.21 | 0.3380, 0.9957 | 1.97, 6.05 |
| Zn | 0.42,1.19 | 0.3280, 1.1283 | 2.06, 6.40 |
| Ga | -0.34, 1.20 | 0.3115, 1.2727 | 2.22, 6.89 |
| Ge | -1.12, 1.21 | 0.2766, 1.4289 | 2.36, 7.17 |
| As | -1.81, 1.21 | 0.2254, 1.5962 | 2.42, 7.20 |
| Se | -2.50, 1.22 | 0.1547, 1.7750 | 2.34, 6.88 |
| Br | -3.19, 1.21 | 0.0581, 1.9650 | 2.17, 6.20 |

## S9. Tabulated values plot

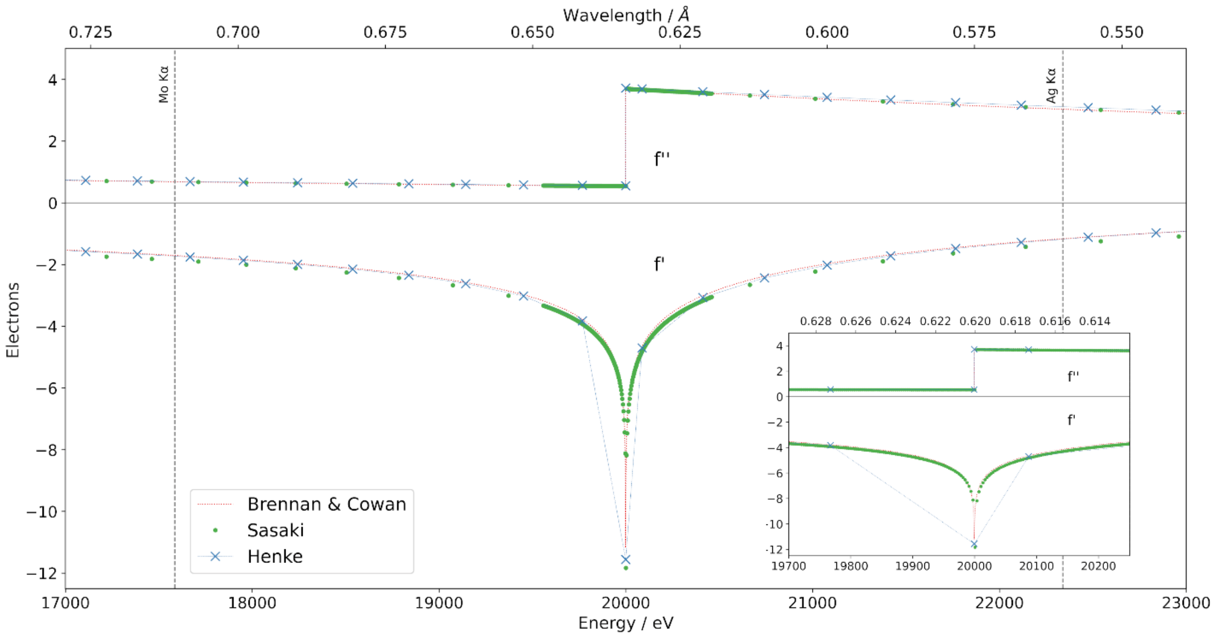


**Fig. S13**: Comparison of the tabulated dispersion values *f´* and *f´´* for Henke *et al.* (1993), Sasaki (1989) and Brennan & Cowan (1992) for molybdenum around its K edge (20,000 eV). Note that values from Henke and Sasaki between the tabulated values are linearly interpolated, whereas Brennan & Cowan directly calculate *f´* and *f´´* for a given energy.

## S10. Henke table data compared to refined anomalous dispersion values

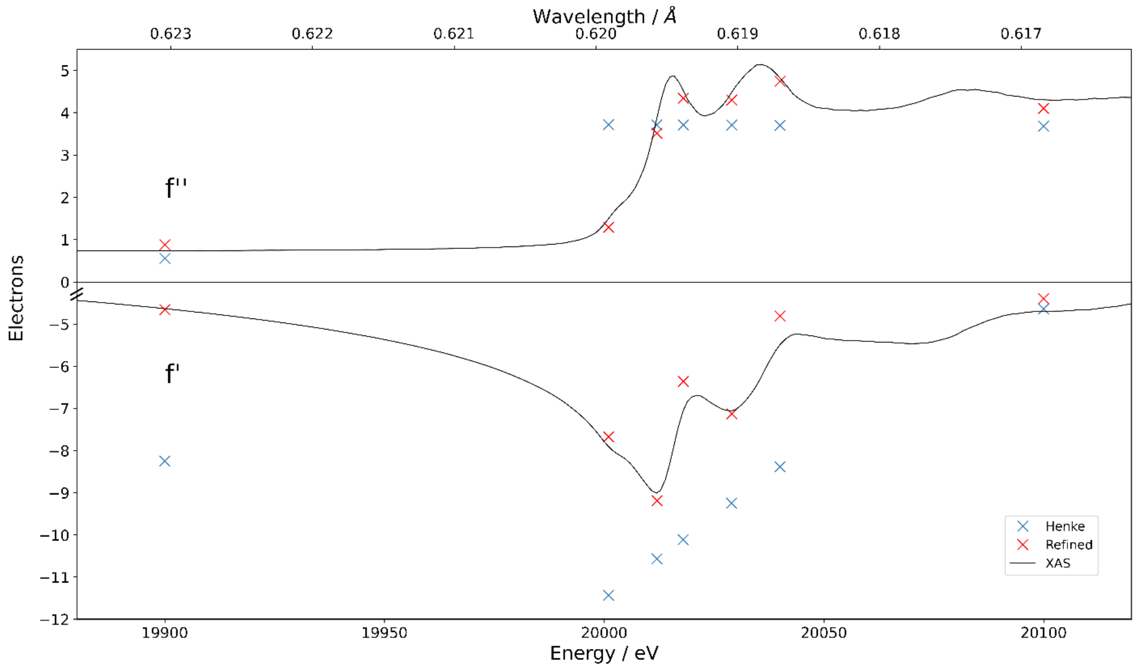


**Fig. S14**: X-ray absorption spectrum of $Mo(CO)_6$ (top black line) and *f´* calculated from it (bottom black line), Henke table (blue, linearly interpolated) and refined values (red) for *f´´* and *f´*.

**S11. Comparison between the independent-atom-model (IAM) and Hirshfeld-atom-refinement (HAR) model**

The refinement of anomalous dispersion parameters can generally be performed with any crystallographic model available in *Olex2*. It should be noted that the choice of the crystallographic model only changes the uncorrected (fully elastic) atomic formfactor $f_0$. The refined anomalous dispersion parameters *f´* and *f´´* are not obtained during e.g. a Hirshfeld-atom-refinement, which in this case was performed using *NoSpherA2* (Kleemiß *et al.*, 2021), but are the results of the subsequent least-squares refinement. As the HAR model provides a more sophisticated method for describing the non-spherical electron density distribution around the atoms, this improved, more sophisticated model was chosen instead of the IAM.

In Fig. S15 the different dispersion values obtained from both HAR and IAM are compared. The general progression of the absolute values is similar. However, while *f´´* shows little difference for the values obtained from the HAR and IAM, the *f´* parameter is systematically lower for the IAM. This suggests that deficiencies of the IAM are compensated either by the HAR approach or an increased dispersion correction.

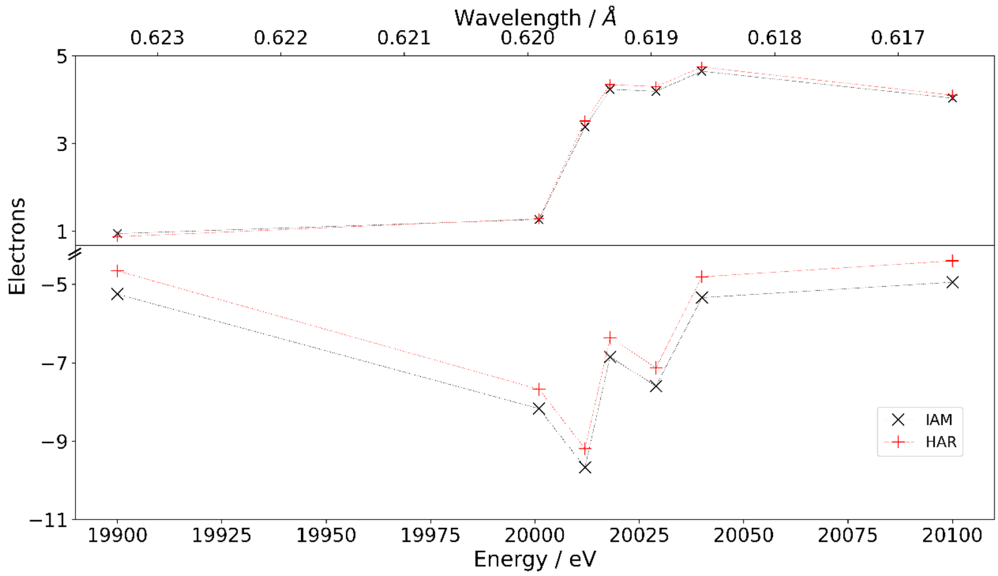


**Fig. S15**: Comparison of the tabulated dispersion values *f´* and *f´´* resulting from the independent-atom-model (black cross) or from a Hirshfeld-atom-refinement model (red plus).

## S12. Correlations of f’ and f’’

**Tab. S9**: Correlations between the anomalous dispersion parameters and the remaining parameters in the full-matrix least-squares refinement

| | 19900 | | 20001 | | 20012 | | 20018 | |
|---|---|---|---|---|---|---|---|---|
| **Parameter** | *f′* | *f′′* | *f′* | *f′′* | *f′* | *f′′* | *f′* | *f′′* |
| Mo01.x | 9.99E-01 | -3.28E-01 | 9.22E-01 | -7.06E-02 | 8.12E-01 | -2.95E-01 | 8.73E-01 | -2.45E-01 |
| Mo01.y | nan | nan | nan | nan | nan | nan | nan | nan |
| Mo01.z | 4.78E-01 | -9.94E-01 | -8.84E-01 | -7.25E-01 | -9.90E-01 | -4.36E-01 | -6.94E-01 | -8.71E-01 |
| Mo01.u11 | 9.97E-01 | -3.10E-01 | 9.98E-01 | 3.79E-01 | 9.96E-01 | 4.00E-01 | 9.94E-01 | 3.60E-01 |
| Mo01.u22 | 9.97E-01 | -3.09E-01 | 9.97E-01 | 3.88E-01 | 9.95E-01 | 4.14E-01 | 9.93E-01 | 3.67E-01 |
| Mo01.u33 | 9.97E-01 | -3.11E-01 | 9.97E-01 | 3.91E-01 | 9.95E-01 | 4.14E-01 | 9.93E-01 | 3.70E-01 |
| Mo01.u12 | nan | nan | nan | nan | nan | nan | nan | nan |
| Mo01.u13 | -5.29E-01 | 9.85E-01 | -7.57E-01 | 3.71E-01 | -2.68E-01 | -9.96E-01 | -4.13E-01 | 7.72E-01 |
| Mo01.u23 | nan | nan | nan | nan | nan | nan | nan | nan |
| Mo01.fp | 1.00E+00 | -3.78E-01 | 1.00E+00 | 3.22E-01 | 1.00E+00 | 3.18E-01 | 1.00E+00 | 2.58E-01 |
| Mo01.fdp | -3.78E-01 | 1.00E+00 | 3.22E-01 | 1.00E+00 | 3.18E-01 | 1.00E+00 | 2.58E-01 | 1.00E+00 |
| O002.x | -5.45E-03 | -9.78E-03 | -1.39E-02 | -1.09E-02 | -1.46E-02 | -1.15E-02 | -1.29E-02 | -1.04E-02 |
| O002.y | -3.49E-03 | -6.32E-03 | -7.72E-03 | -5.95E-03 | -6.04E-03 | -4.68E-03 | -7.46E-03 | -6.06E-03 |
| O002.z | -6.25E-03 | -1.12E-02 | -1.44E-02 | -1.13E-02 | -1.54E-02 | -1.22E-02 | -1.41E-02 | -1.15E-02 |
| O002.u11 | -1.80E-02 | -3.20E-02 | -2.38E-02 | -1.86E-02 | -1.14E-02 | -8.97E-03 | -1.37E-02 | -1.10E-02 |
| O002.u22 | -7.00E-03 | -1.24E-02 | -1.75E-02 | -1.37E-02 | -2.42E-02 | -1.90E-02 | -2.81E-02 | -2.27E-02 |
| O002.u33 | -1.53E-02 | -2.73E-02 | -2.10E-02 | -1.64E-02 | -2.06E-02 | -1.61E-02 | -2.03E-02 | -1.65E-02 |
| O002.u12 | -2.21E-03 | -3.71E-03 | 5.85E-03 | 4.56E-03 | 1.42E-02 | 1.12E-02 | 7.46E-03 | 6.08E-03 |
| O002.u13 | -2.94E-04 | -4.90E-04 | 4.30E-03 | 3.29E-03 | -3.10E-03 | -2.35E-03 | -5.06E-03 | -4.09E-03 |
| O002.u23 | -7.24E-03 | -1.28E-02 | -1.76E-02 | -1.37E-02 | -1.50E-02 | -1.16E-02 | -1.60E-02 | -1.30E-02 |
| O003.x | -4.11E-03 | -7.31E-03 | -1.18E-02 | -9.18E-03 | -1.44E-02 | -1.12E-02 | -1.28E-02 | -1.03E-02 |
| O003.y | nan | nan | nan | nan | nan | nan | nan | nan |
| O003.z | -6.66E-03 | -1.21E-02 | -1.83E-02 | -1.42E-02 | -1.92E-02 | -1.49E-02 | -1.73E-02 | -1.40E-02 |
| O003.u11 | -1.26E-02 | -2.25E-02 | -3.83E-02 | -2.99E-02 | -3.79E-02 | -2.96E-02 | -3.16E-02 | -2.55E-02 |
| O003.u22 | -4.69E-03 | -8.31E-03 | -2.13E-02 | -1.67E-02 | -1.47E-02 | -1.16E-02 | -1.14E-02 | -9.22E-03 |
| O003.u33 | -2.35E-03 | -3.94E-03 | 4.77E-03 | 3.70E-03 | 5.37E-03 | 4.11E-03 | 8.11E-03 | 6.57E-03 |
| O003.u12 | nan | nan | nan | nan | nan | nan | nan | nan |
| O003.u13 | -5.33E-03 | -9.39E-03 | -3.96E-04 | -4.25E-04 | -6.19E-03 | -4.88E-03 | -8.24E-03 | -6.69E-03 |
| O003.u23 | nan | nan | nan | nan | nan | nan | nan | nan |
| O004.x | -5.43E-03 | -9.60E-03 | -1.30E-02 | -1.02E-02 | -1.34E-02 | -1.05E-02 | -1.36E-02 | -1.09E-02 |
| O004.y | nan | nan | nan | nan | nan | nan | nan | nan |
| O004.z | -7.95E-03 | -1.40E-02 | -2.06E-02 | -1.59E-02 | -2.13E-02 | -1.66E-02 | -2.00E-02 | -1.61E-02 |
| O004.u11 | 5.34E-03 | 9.83E-03 | 3.10E-03 | 2.39E-03 | 9.94E-04 | 7.35E-04 | 8.63E-03 | 7.01E-03 |
| O004.u22 | -9.29E-03 | -1.68E-02 | -2.30E-02 | -1.79E-02 | -1.65E-02 | -1.29E-02 | -1.75E-02 | -1.41E-02 |
| O004.u33 | -9.25E-03 | -1.64E-02 | -2.48E-02 | -1.93E-02 | -2.16E-02 | -1.69E-02 | -1.87E-02 | -1.51E-02 |
| O004.u12 | nan | nan | nan | nan | nan | nan | nan | nan |
| O004.u13 | -1.07E-02 | -1.91E-02 | -2.45E-02 | -1.91E-02 | -2.16E-02 | -1.69E-02 | -2.16E-02 | -1.74E-02 |
| O004.u23 | nan | nan | nan | nan | nan | nan | nan | nan |
| O005.x | -7.86E-03 | -1.41E-02 | -2.14E-02 | -1.67E-02 | -2.09E-02 | -1.64E-02 | -2.06E-02 | -1.66E-02 |
| O005.y | -8.37E-03 | -1.49E-02 | -1.98E-02 | -1.55E-02 | -1.81E-02 | -1.42E-02 | -1.81E-02 | -1.46E-02 |
| O005.z | -7.14E-03 | -1.30E-02 | -1.94E-02 | -1.50E-02 | -1.98E-02 | -1.54E-02 | -1.80E-02 | -1.47E-02 |

| | | | | | | | | |
|---|---|---|---|---|---|---|---|---|
| O005.u11 | -1.25E-02 | -2.25E-02 | -2.07E-02 | -1.61E-02 | -3.37E-02 | -2.63E-02 | -3.31E-02 | -2.68E-02 |
| O005.u22 | -5.18E-03 | -9.18E-03 | -1.59E-02 | -1.25E-02 | -2.29E-03 | -1.88E-03 | 1.69E-04 | 1.54E-04 |
| O005.u33 | -3.70E-03 | -6.39E-03 | -1.12E-02 | -8.79E-03 | -2.38E-02 | -1.86E-02 | -1.23E-02 | -9.97E-03 |
| O005.u12 | -1.52E-03 | -2.58E-03 | -7.20E-03 | -5.60E-03 | -1.66E-02 | -1.29E-02 | -1.08E-02 | -8.63E-03 |
| O005.u13 | -6.96E-03 | -1.23E-02 | -1.17E-02 | -9.07E-03 | -5.85E-03 | -4.50E-03 | -3.64E-04 | -2.76E-04 |
| O005.u23 | -9.94E-03 | -1.80E-02 | -5.86E-03 | -4.58E-03 | -7.23E-03 | -5.64E-03 | -5.31E-03 | -4.34E-03 |
| C006.x | -4.83E-03 | -8.65E-03 | -1.15E-02 | -8.94E-03 | -1.19E-02 | -9.32E-03 | -1.07E-02 | -8.60E-03 |
| C006.y | -5.40E-03 | -9.67E-03 | -1.20E-02 | -9.42E-03 | -1.31E-02 | -1.02E-02 | -1.35E-02 | -1.09E-02 |
| C006.z | -5.87E-03 | -1.04E-02 | -1.29E-02 | -1.01E-02 | -1.36E-02 | -1.07E-02 | -1.31E-02 | -1.06E-02 |
| C006.u11 | 5.41E-03 | 9.69E-03 | -1.54E-02 | -1.21E-02 | -1.67E-02 | -1.31E-02 | -1.38E-03 | -1.13E-03 |
| C006.u22 | -1.04E-02 | -1.86E-02 | -3.05E-02 | -2.38E-02 | -2.30E-02 | -1.80E-02 | -2.28E-02 | -1.84E-02 |
| C006.u33 | -1.36E-02 | -2.42E-02 | -2.80E-02 | -2.18E-02 | -2.31E-02 | -1.81E-02 | -2.66E-02 | -2.15E-02 |
| C006.u12 | -6.36E-03 | -1.14E-02 | -9.04E-03 | -7.00E-03 | -1.04E-02 | -8.11E-03 | -8.73E-03 | -7.01E-03 |
| C006.u13 | -9.69E-03 | -1.75E-02 | -6.88E-03 | -5.35E-03 | -3.45E-03 | -2.64E-03 | -2.98E-03 | -2.44E-03 |
| C006.u23 | -8.67E-03 | -1.56E-02 | -2.49E-02 | -1.93E-02 | -2.73E-02 | -2.13E-02 | -2.43E-02 | -1.96E-02 |
| C007.x | -5.19E-03 | -9.13E-03 | -1.19E-02 | -9.21E-03 | -1.08E-02 | -8.39E-03 | -1.05E-02 | -8.42E-03 |
| C007.y | nan | nan | nan | nan | nan | nan | nan | nan |
| C007.z | -5.69E-03 | -1.01E-02 | -1.32E-02 | -1.03E-02 | -1.36E-02 | -1.07E-02 | -1.29E-02 | -1.04E-02 |
| C007.u11 | 1.09E-04 | 1.99E-04 | -8.78E-03 | -6.91E-03 | -3.07E-03 | -2.51E-03 | 2.61E-03 | 2.10E-03 |
| C007.u22 | 1.89E-03 | 3.41E-03 | -2.31E-03 | -1.77E-03 | 3.74E-03 | 2.91E-03 | 6.16E-03 | 4.98E-03 |
| C007.u33 | -1.17E-02 | -2.09E-02 | -2.89E-02 | -2.25E-02 | -2.79E-02 | -2.17E-02 | -2.67E-02 | -2.16E-02 |
| C007.u12 | nan | nan | nan | nan | nan | nan | nan | nan |
| C007.u13 | -5.35E-03 | -9.56E-03 | -1.66E-02 | -1.29E-02 | -2.77E-02 | -2.16E-02 | -2.26E-02 | -1.83E-02 |
| C007.u23 | nan | nan | nan | nan | nan | nan | nan | nan |
| C008.x | -7.15E-03 | -1.29E-02 | -1.68E-02 | -1.32E-02 | -1.70E-02 | -1.34E-02 | -1.65E-02 | -1.34E-02 |
| C008.y | nan | nan | nan | nan | nan | nan | nan | nan |
| C008.z | -6.91E-03 | -1.26E-02 | -1.66E-02 | -1.28E-02 | -1.66E-02 | -1.29E-02 | -1.60E-02 | -1.29E-02 |
| C008.u11 | 2.47E-03 | 4.41E-03 | 2.60E-03 | 2.00E-03 | 9.85E-04 | 7.17E-04 | 7.55E-05 | 3.78E-05 |
| C008.u22 | -5.06E-03 | -9.03E-03 | -1.31E-02 | -1.03E-02 | -1.95E-02 | -1.53E-02 | -1.71E-02 | -1.38E-02 |
| C008.u33 | -8.97E-03 | -1.60E-02 | -2.07E-02 | -1.62E-02 | -1.13E-02 | -8.89E-03 | -1.25E-02 | -1.01E-02 |
| C008.u12 | nan | nan | nan | nan | nan | nan | nan | nan |
| C008.u13 | -3.96E-03 | -7.10E-03 | -7.49E-03 | -5.90E-03 | -9.51E-03 | -7.45E-03 | -8.32E-03 | -6.74E-03 |
| C008.u23 | nan | nan | nan | nan | nan | nan | nan | nan |
| C009.x | -6.83E-03 | -1.22E-02 | -1.60E-02 | -1.25E-02 | -1.60E-02 | -1.25E-02 | -1.55E-02 | -1.25E-02 |
| C009.y | -7.13E-03 | -1.29E-02 | -1.62E-02 | -1.26E-02 | -1.63E-02 | -1.28E-02 | -1.58E-02 | -1.28E-02 |
| C009.z | -6.86E-03 | -1.22E-02 | -1.56E-02 | -1.21E-02 | -1.56E-02 | -1.21E-02 | -1.51E-02 | -1.21E-02 |
| C009.u11 | -1.78E-03 | -3.20E-03 | -4.97E-03 | -3.88E-03 | -4.77E-03 | -3.73E-03 | -4.98E-03 | -4.02E-03 |
| C009.u22 | -3.38E-03 | -6.06E-03 | -7.99E-03 | -6.24E-03 | -7.85E-03 | -6.14E-03 | -6.98E-03 | -5.64E-03 |
| C009.u33 | -6.96E-03 | -1.24E-02 | -1.59E-02 | -1.24E-02 | -1.58E-02 | -1.24E-02 | -1.53E-02 | -1.24E-02 |
| C009.u12 | -6.99E-03 | -1.25E-02 | -1.56E-02 | -1.22E-02 | -1.55E-02 | -1.21E-02 | -1.50E-02 | -1.22E-02 |
| C009.u13 | -6.72E-03 | -1.20E-02 | -1.53E-02 | -1.20E-02 | -1.52E-02 | -1.19E-02 | -1.47E-02 | -1.19E-02 |
| C009.u23 | -6.81E-03 | -1.22E-02 | -1.56E-02 | -1.22E-02 | -1.56E-02 | -1.22E-02 | -1.51E-02 | -1.22E-02 |

| | 20029 | | 20045 | | 20100 | |
|---|---|---|---|---|---|---|
| **Parameter** | *f′* | *f′′* | *f′* | *f′′* | *f′* | *f′′* |
| Mo01.x | 8.81E-01 | -1.98E-01 | 8.68E-01 | -3.25E-01 | 9.21E-01 | -2.78E-01 |
| Mo01.y | nan | nan | nan | nan | nan | nan |
| Mo01.z | -1.27E-01 | -9.82E-01 | -6.55E-01 | 6.16E-01 | -9.78E-01 | 8.40E-02 |
| Mo01.u11 | 9.95E-01 | 3.80E-01 | 9.92E-01 | 3.11E-01 | 9.94E-01 | 2.31E-01 |
| Mo01.u22 | 9.95E-01 | 3.88E-01 | 9.91E-01 | 3.21E-01 | 9.93E-01 | 2.39E-01 |
| Mo01.u33 | 9.94E-01 | 3.92E-01 | 9.90E-01 | 3.23E-01 | 9.92E-01 | 2.43E-01 |
| Mo01.u12 | nan | nan | nan | nan | nan | nan |
| Mo01.u13 | -8.89E-01 | 1.80E-01 | -4.98E-01 | 7.58E-01 | -4.24E-01 | 8.49E-01 |
| Mo01.u23 | nan | nan | nan | nan | nan | nan |
| Mo01.fp | 1.00E+00 | 2.90E-01 | 1.00E+00 | 1.88E-01 | 1.00E+00 | 1.19E-01 |
| Mo01.fdp | 2.90E-01 | 1.00E+00 | 1.88E-01 | 1.00E+00 | 1.19E-01 | 1.00E+00 |
| O002.x | -1.30E-02 | -1.03E-02 | -1.23E-02 | -1.04E-02 | -1.09E-02 | -9.73E-03 |
| O002.y | -7.52E-03 | -5.98E-03 | -7.51E-03 | -6.41E-03 | -7.03E-03 | -6.35E-03 |
| O002.z | -1.44E-02 | -1.16E-02 | -1.30E-02 | -1.11E-02 | -1.24E-02 | -1.10E-02 |
| O002.u11 | -1.14E-02 | -9.03E-03 | -1.95E-02 | -1.63E-02 | -2.30E-02 | -2.03E-02 |
| O002.u22 | -2.48E-02 | -1.97E-02 | -2.87E-02 | -2.42E-02 | -3.01E-02 | -2.68E-02 |
| O002.u33 | -1.72E-02 | -1.37E-02 | -2.39E-02 | -2.03E-02 | -2.10E-02 | -1.88E-02 |
| O002.u12 | 8.74E-03 | 6.99E-03 | 5.45E-04 | 5.56E-04 | 7.89E-04 | 8.01E-04 |
| O002.u13 | 3.03E-03 | 2.39E-03 | -2.09E-03 | -1.69E-03 | -4.51E-03 | -3.92E-03 |
| O002.u23 | -1.75E-02 | -1.39E-02 | -1.19E-02 | -1.00E-02 | -1.06E-02 | -9.40E-03 |
| O003.x | -1.28E-02 | -1.02E-02 | -1.17E-02 | -9.80E-03 | -1.09E-02 | -9.68E-03 |
| O003.y | nan | nan | nan | nan | nan | nan |
| O003.z | -1.88E-02 | -1.49E-02 | -1.56E-02 | -1.32E-02 | -1.51E-02 | -1.35E-02 |
| O003.u11 | -3.12E-02 | -2.48E-02 | -2.81E-02 | -2.37E-02 | -2.98E-02 | -2.65E-02 |
| O003.u22 | -1.19E-02 | -9.42E-03 | -8.92E-03 | -7.44E-03 | -7.43E-03 | -6.51E-03 |
| O003.u33 | 6.93E-03 | 5.48E-03 | 6.94E-03 | 5.94E-03 | 8.18E-03 | 7.39E-03 |
| O003.u12 | nan | nan | nan | nan | nan | nan |
| O003.u13 | -2.40E-03 | -1.97E-03 | -1.29E-02 | -1.10E-02 | -1.20E-02 | -1.07E-02 |
| O003.u23 | nan | nan | nan | nan | nan | nan |
| O004.x | -1.40E-02 | -1.11E-02 | -1.36E-02 | -1.14E-02 | -1.27E-02 | -1.12E-02 |
| O004.y | nan | nan | nan | nan | nan | nan |
| O004.z | -2.11E-02 | -1.67E-02 | -1.87E-02 | -1.58E-02 | -1.78E-02 | -1.58E-02 |
| O004.u11 | 1.47E-03 | 1.18E-03 | 1.09E-02 | 9.32E-03 | 4.55E-03 | 4.17E-03 |
| O004.u22 | -1.47E-02 | -1.17E-02 | -1.72E-02 | -1.46E-02 | -2.06E-02 | -1.84E-02 |
| O004.u33 | -1.78E-02 | -1.42E-02 | -1.51E-02 | -1.28E-02 | -1.52E-02 | -1.35E-02 |
| O004.u12 | nan | nan | nan | nan | nan | nan |
| O004.u13 | -2.13E-02 | -1.69E-02 | -2.07E-02 | -1.75E-02 | -1.68E-02 | -1.49E-02 |
| O004.u23 | nan | nan | nan | nan | nan | nan |
| O005.x | -1.94E-02 | -1.54E-02 | -1.89E-02 | -1.60E-02 | -1.79E-02 | -1.60E-02 |
| O005.y | -1.88E-02 | -1.49E-02 | -1.78E-02 | -1.50E-02 | -1.70E-02 | -1.51E-02 |
| O005.z | -1.96E-02 | -1.56E-02 | -1.69E-02 | -1.45E-02 | -1.63E-02 | -1.47E-02 |
| O005.u11 | -4.23E-02 | -3.36E-02 | -3.24E-02 | -2.75E-02 | -2.87E-02 | -2.57E-02 |
| O005.u22 | -2.37E-04 | -2.05E-04 | -1.10E-04 | -3.20E-05 | -1.08E-03 | -8.76E-04 |
| O005.u33 | -1.25E-02 | -9.93E-03 | -8.85E-03 | -7.47E-03 | -8.59E-03 | -7.61E-03 |
| O005.u12 | -1.45E-02 | -1.15E-02 | -9.02E-03 | -7.55E-03 | -5.63E-03 | -4.94E-03 |
| O005.u13 | -6.34E-04 | -4.77E-04 | -6.19E-03 | -5.17E-03 | 3.81E-03 | 3.50E-03 |

| | | | | | | |
|---|---|---|---|---|---|---|
| O005.u23 | -2.56E-03 | -2.05E-03 | -6.05E-03 | -5.17E-03 | -5.89E-03 | -5.36E-03 |
| C006.x | -1.12E-02 | -8.85E-03 | -9.77E-03 | -8.28E-03 | -8.52E-03 | -7.59E-03 |
| C006.y | -1.30E-02 | -1.03E-02 | -1.33E-02 | -1.12E-02 | -1.25E-02 | -1.11E-02 |
| C006.z | -1.36E-02 | -1.08E-02 | -1.24E-02 | -1.05E-02 | -1.23E-02 | -1.10E-02 |
| C006.u11 | 3.08E-03 | 2.41E-03 | 6.13E-03 | 5.18E-03 | 5.12E-03 | 4.62E-03 |
| C006.u22 | -2.31E-02 | -1.84E-02 | -2.15E-02 | -1.83E-02 | -2.12E-02 | -1.89E-02 |
| C006.u33 | -2.27E-02 | -1.81E-02 | -2.43E-02 | -2.06E-02 | -2.64E-02 | -2.35E-02 |
| C006.u12 | -7.49E-03 | -5.89E-03 | -4.95E-03 | -4.16E-03 | -7.54E-03 | -6.74E-03 |
| C006.u13 | -2.54E-03 | -2.02E-03 | -5.31E-03 | -4.52E-03 | -3.27E-03 | -2.99E-03 |
| C006.u23 | -2.29E-02 | -1.81E-02 | -2.40E-02 | -2.04E-02 | -1.99E-02 | -1.77E-02 |
| C007.x | -1.10E-02 | -8.60E-03 | -1.03E-02 | -8.58E-03 | -9.80E-03 | -8.59E-03 |
| C007.y | nan | nan | nan | nan | nan | nan |
| C007.z | -1.32E-02 | -1.04E-02 | -1.23E-02 | -1.04E-02 | -1.18E-02 | -1.05E-02 |
| C007.u11 | 5.27E-03 | 4.14E-03 | 4.79E-03 | 4.08E-03 | 3.69E-03 | 3.36E-03 |
| C007.u22 | 3.76E-03 | 2.99E-03 | 6.82E-03 | 5.76E-03 | 6.21E-04 | 5.28E-04 |
| C007.u33 | -2.72E-02 | -2.16E-02 | -2.54E-02 | -2.15E-02 | -2.44E-02 | -2.18E-02 |
| C007.u12 | nan | nan | nan | nan | nan | nan |
| C007.u13 | -2.72E-02 | -2.16E-02 | -2.02E-02 | -1.71E-02 | -1.79E-02 | -1.59E-02 |
| C007.u23 | nan | nan | nan | nan | nan | nan |
| C008.x | -1.71E-02 | -1.36E-02 | -1.58E-02 | -1.33E-02 | -1.49E-02 | -1.33E-02 |
| C008.y | nan | nan | nan | nan | nan | nan |
| C008.z | -1.65E-02 | -1.31E-02 | -1.52E-02 | -1.29E-02 | -1.46E-02 | -1.31E-02 |
| C008.u11 | 4.65E-03 | 3.66E-03 | 1.50E-03 | 1.25E-03 | 4.37E-03 | 3.88E-03 |
| C008.u22 | -1.77E-02 | -1.41E-02 | -1.50E-02 | -1.27E-02 | -1.56E-02 | -1.39E-02 |
| C008.u33 | -1.09E-02 | -8.65E-03 | -1.30E-02 | -1.10E-02 | -1.33E-02 | -1.18E-02 |
| C008.u12 | nan | nan | nan | nan | nan | nan |
| C008.u13 | -7.59E-03 | -6.04E-03 | -9.82E-03 | -8.32E-03 | -8.61E-03 | -7.66E-03 |
| C008.u23 | nan | nan | nan | nan | nan | nan |
| C009.x | -1.58E-02 | -1.25E-02 | -1.48E-02 | -1.25E-02 | -1.39E-02 | -1.24E-02 |
| C009.y | -1.60E-02 | -1.27E-02 | -1.52E-02 | -1.29E-02 | -1.43E-02 | -1.28E-02 |
| C009.z | -1.53E-02 | -1.21E-02 | -1.44E-02 | -1.22E-02 | -1.37E-02 | -1.22E-02 |
| C009.u11 | -5.39E-03 | -4.28E-03 | -4.39E-03 | -3.72E-03 | -3.93E-03 | -3.50E-03 |
| C009.u22 | -7.30E-03 | -5.80E-03 | -6.51E-03 | -5.51E-03 | -6.15E-03 | -5.48E-03 |
| C009.u33 | -1.56E-02 | -1.24E-02 | -1.47E-02 | -1.24E-02 | -1.39E-02 | -1.24E-02 |
| C009.u12 | -1.54E-02 | -1.23E-02 | -1.46E-02 | -1.24E-02 | -1.38E-02 | -1.23E-02 |
| C009.u13 | -1.49E-02 | -1.19E-02 | -1.41E-02 | -1.20E-02 | -1.34E-02 | -1.20E-02 |
| C009.u23 | -1.53E-02 | -1.22E-02 | -1.44E-02 | -1.22E-02 | -1.37E-02 | -1.22E-02 |

Significant correlations (> 0.05) between the anomalous dispersion parameters and the other parameters in the least-squares refinement were only found for the atomic positional and displacement parameters for molybdenum. Both, anomalous dispersion as well as atomic displacement parameters, which themselves are closely connected to the positional parameters, alter the modelled electron density in proximity of an atom. Therefore, a strong correlation between the atomic parameters of an atom and its dispersion parameters is expected. This finding is also in good agreement with the very small displacement parameters for e.g., the model based on the Sasaki table at 20,001 eV, when the table assumes that the energy is already sufficient to excite the resonance frequencies for the K-edge, and therefore subtracts more electrons from the molybdenum form factor than physically needed.

The other parameters however show no significant correlation with the anomalous dispersion parameters in these experiments.

## S13. Leverage analysis

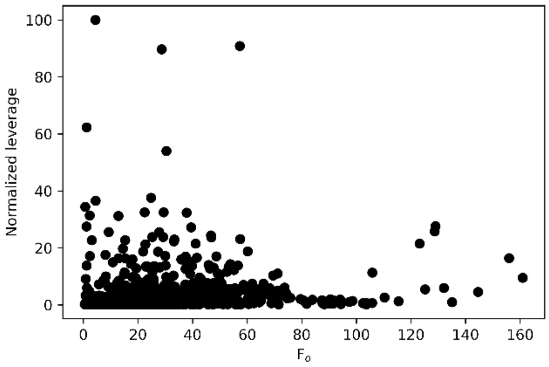


**19,900 eV**, Mo01.fp

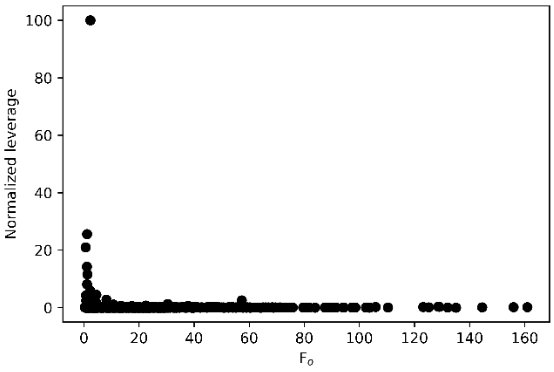


**19,900 eV**, Mo01.fdp

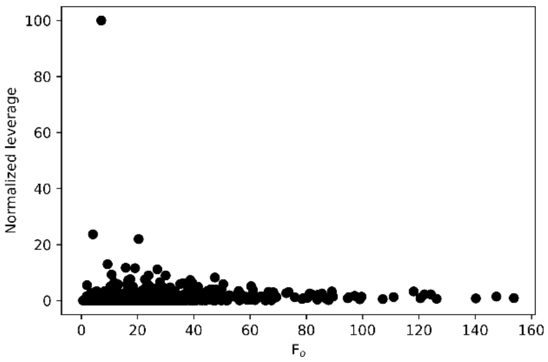


**20,001 eV**, Mo01.fp

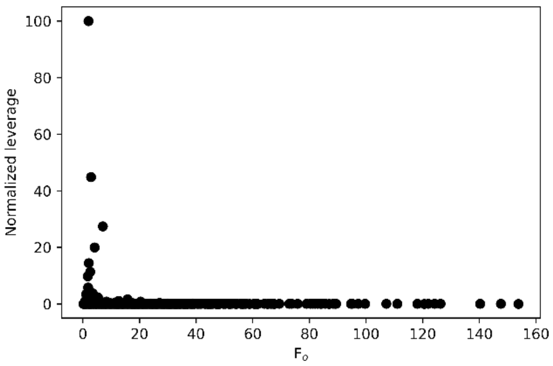


**20,001 eV**, Mo01.fdp

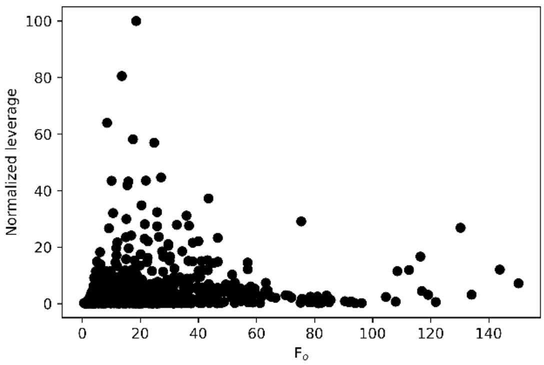


**20,012 eV**, Mo01.fp

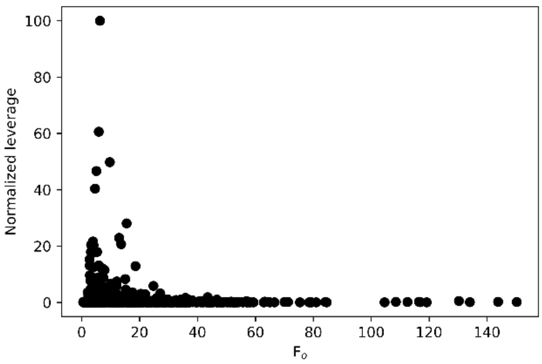


**20,012 eV**, Mo01.fdp

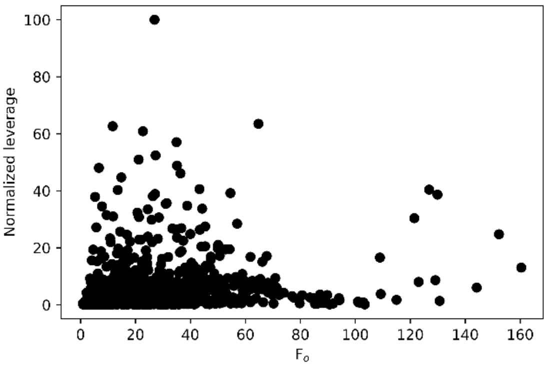


**20,018 eV**, Mo01.fp

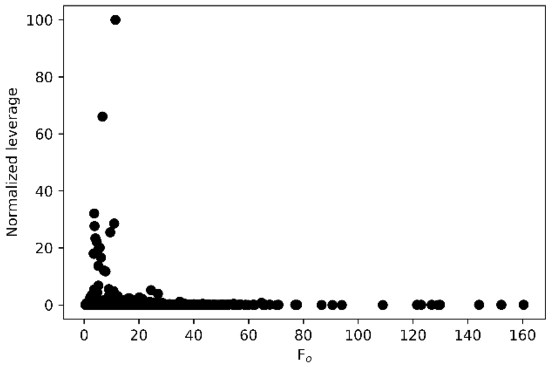


**20,018 eV**, Mo01.fdp

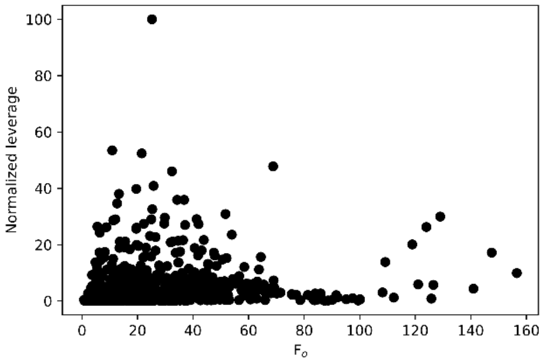


**20,029 eV**, Mo01.fp

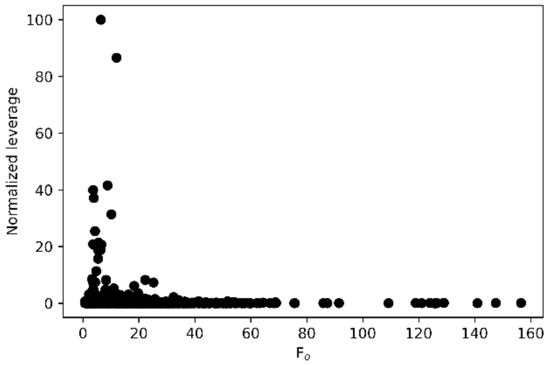


**20,029 eV**, Mo01.fdp

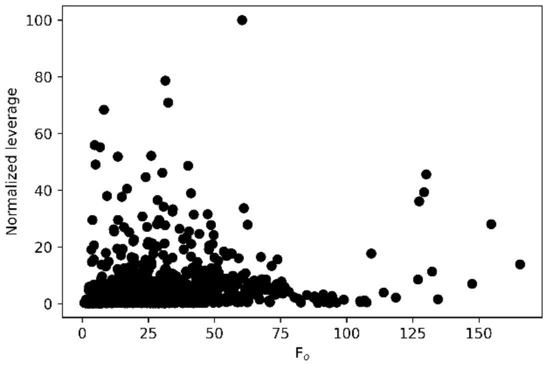


**20,040 eV**, Mo01.fp

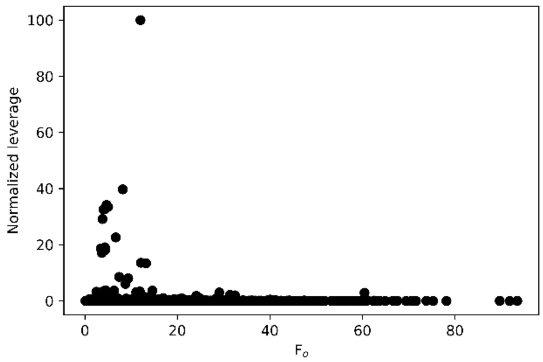


**20,040 eV**, Mo01.fdp

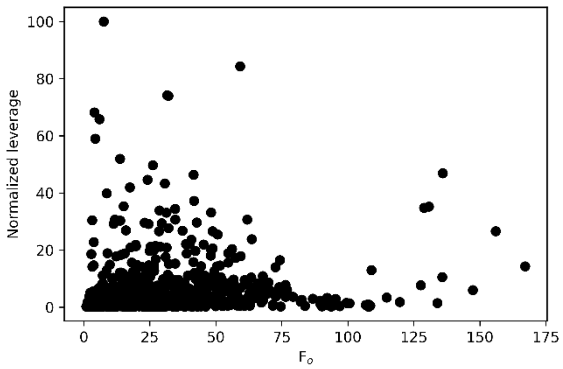


**20,100 eV**, Mo01.fp

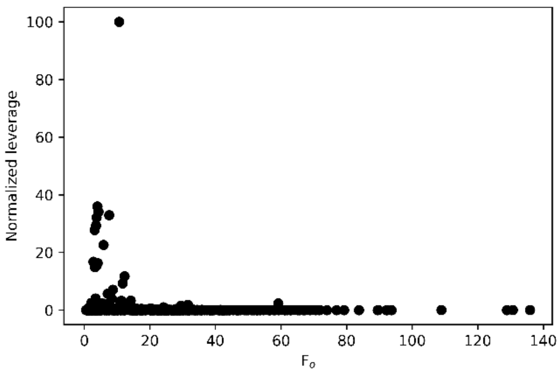


**20,100 eV**, Mo01.fdp

**Fig. S16**: Scatterplot for the leverage of each reflection of the 1000 most influential reflections on *f´* (left) and *f´´* (right) for the dispersive (Mo01.fp) and absorptive (Mo01.fdp) part of the anomalous dispersion correction for each energy according to Parsons *et al.* (2012) plotted against their observed intensity. The strongest influential reflection was normalized to a leverage of 100 and each subsequent reflection was normalized as a fraction of the highest leverage × 100.

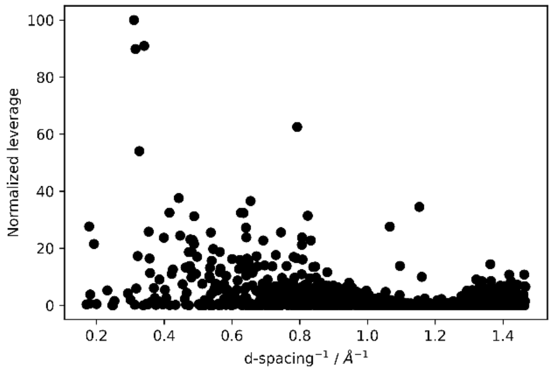


**19,900 eV**, Mo01.fp

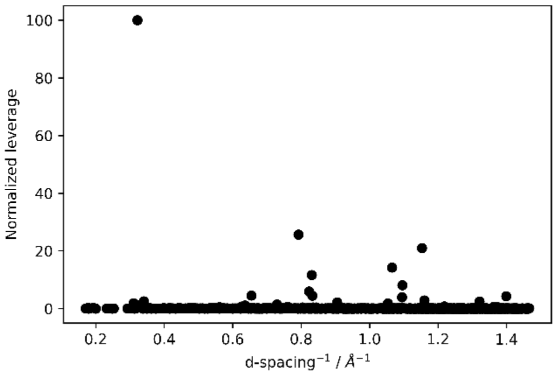


**19,900 eV**, Mo01.fdp

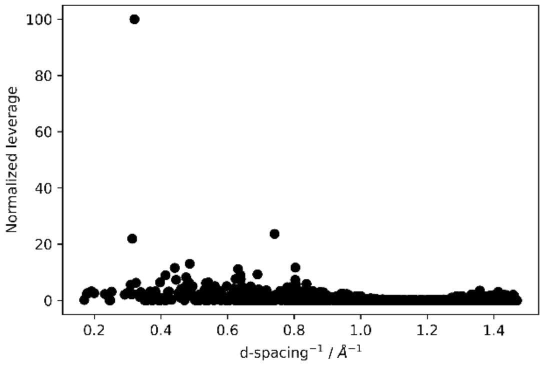


**20,001 eV**, Mo01.fp

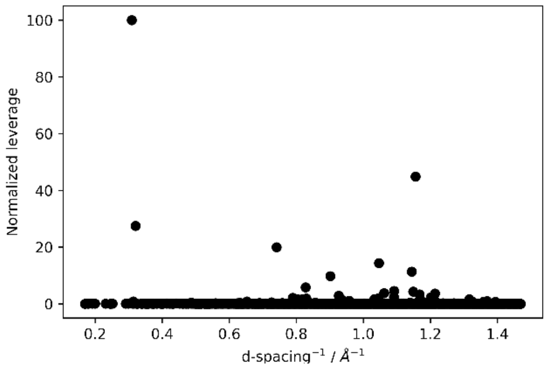


**20,001 eV**, Mo01.fp

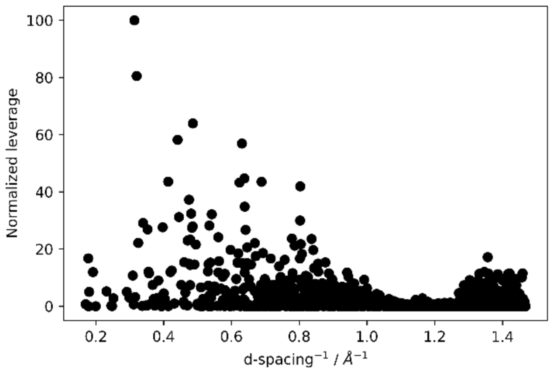


**20,012 eV**, Mo01.fp

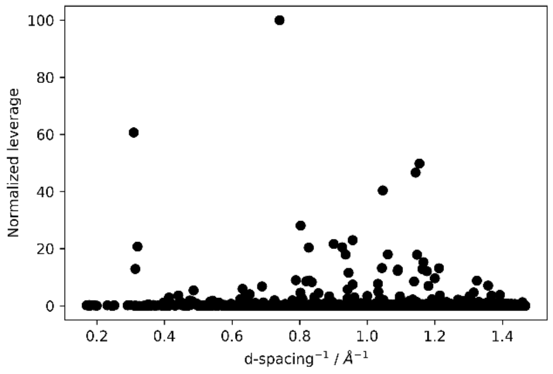


**20,012 eV**, Mo01.fp

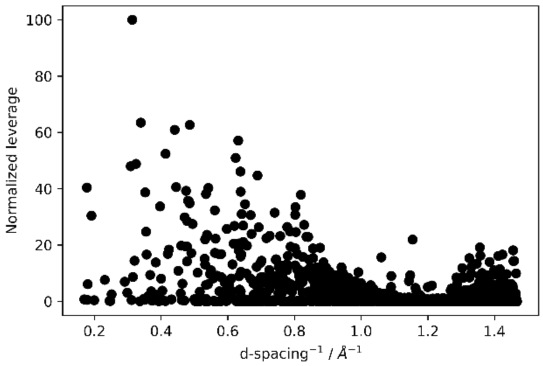


**20,018 eV**, Mo01.fp

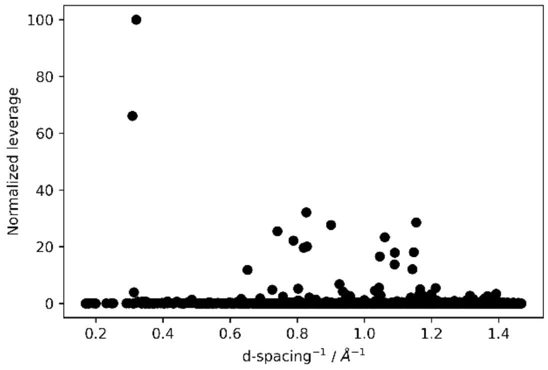


**20,018 eV**, Mo01.fp

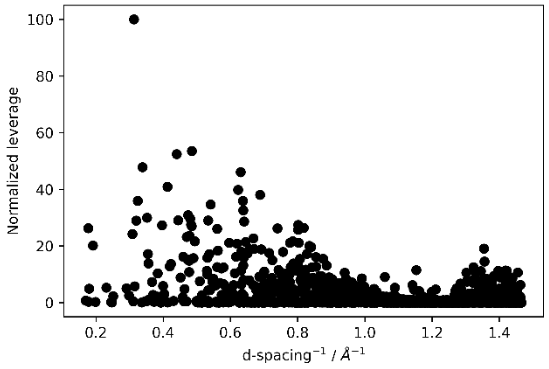


**20,029 eV**, Mo01.fp

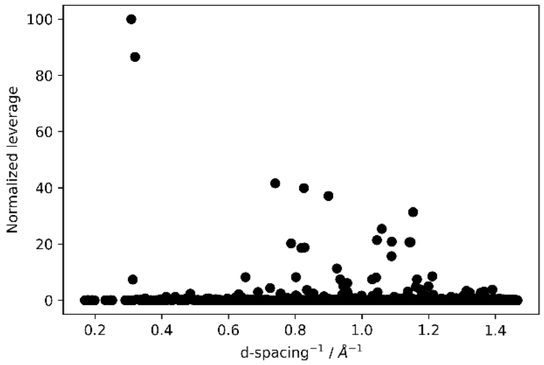


**20,029 eV**, Mo01.fp

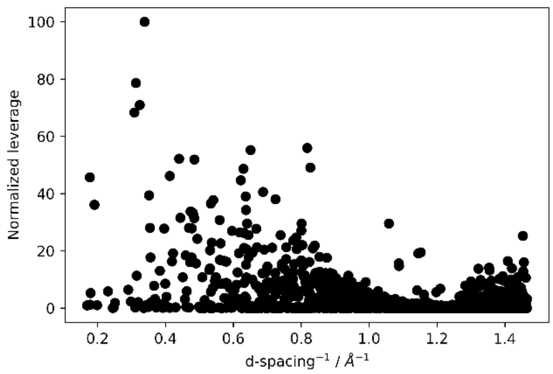


**20,040 eV**, Mo01.fp

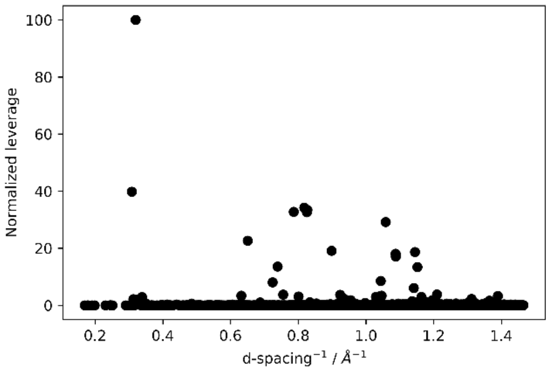


**20,040 eV**, Mo01.fp

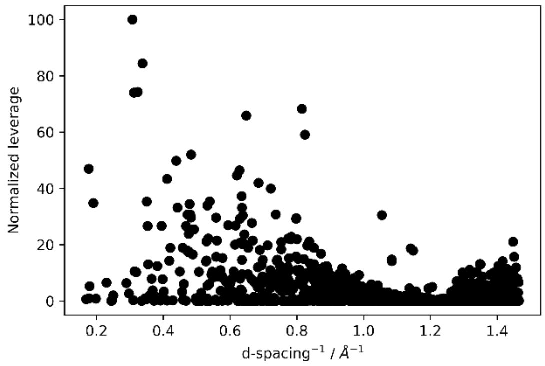


**20,100 eV**, Mo01.fp

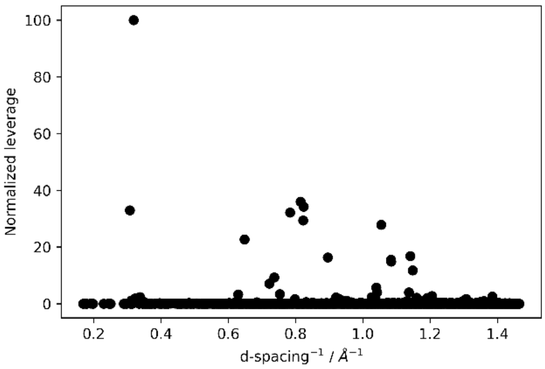


**20,100 eV**, Mo01.fp

**Fig. S17**: Scatterplot for the leverage of each reflection of the 1000 most influential reflections on *f´* (left) and *f´´* (right) for the dispersive (Mo01.fp) and absorptive (Mo01.fdp) part of the anomalous dispersion correction for each energy according to Parsons *et al.* (2012) plotted against their inverse *d*-spacing. The strongest influential reflection was normalized to a leverage of 100 and each subsequent reflection was normalized as a fraction of the highest leverage × 100.

The leverage analysis shows that *f´´* is generally influenced by only few reflections, which are of low intensity and medium high resolution (0.6 – 1.2 Å$^{-1}$). This is consistent with the decreasing valence contribution to scattering at high resolution. The core part determines the photo-absorption. Also, *f´* is more affected at lower intensity, as well as at low resolution.

The missing reflections in the four data sets at 19.900, 20.029, 20.041 and 20.100 eV were checked for their effect on the refined values of *f´* and *f´´*, but it is negligible in all cases.

## S14. Errors of *f´* and *f´´* at laboratory diffractometers

To determine a standard deviation for the refined values of the anomalous dispersion parameters, six high-quality diffraction experiments of **1** were performed using in-house X-ray sources with molybdenum (Rigaku Oxford Diffraction Synergy-DW) as well as silver radiation (Rigaku Oxford Diffraction SuperNova). Each measurement was performed at 123 K. Mo Kα ($E_{K\alpha1,2}$ = 17,444 eV) lies

far below and Ag Kα ($E_{K\alpha1,2}$ = 22,105 eV) above the absorption edge of molybdenum (20,000 eV). A Hirshfeld-Atom-Refinement with PBE0/Jorge-TZP-DKH level of theory was performed for each dataset. Ag data sets were refined up to a resolution of 0.55 Å and Mo data sets up to 0.65 Å resolution. Every data set was treated with multi-scan absorption correction.

**Tab. S10**: Results of the dispersion refinements of 1 using Mo Kα and Ag Kα radiation compared to the values by Sasaki (1989).

| | Mo Kα | | Ag Kα | |
|---|---|---|---|---|
| Nr. | *f´* | *f´´* | *f´* | *f´´* |
| 1 | -1.46(4) | 1.63(11) | -1.22(8) | 3.19(19) |
| 2 | -1.41(3) | 1.72(11) | -1.24(7) | 3.68(10) |
| 3 | -1.36(3) | 1.43(11) | -1.07(5) | 3.35(10) |
| 4 | -1.35(3) | 1.53(12) | -1.24(5) | 3.57(8) |
| 5 | -1.43(4) | 1.53(10) | -1.14(5) | 3.90(7) |
| 6 | -1.36(4) | 1.46(11) | -0.91(5) | 3.61(4) |
| Sasaki table | -1.8153 | 0.6883 | -1.4447 | 3.1114 |

Tab. S10 shows the resulting refined anomalous dispersion parameters for each measurement. The crystallographic information files as well as the evaluation (spreadsheet) are supplemented as zip archive.

The standard uncertainty over six measurements for Mo Kα radiation *f´* ±0.04, *f´´* ±0.08 and for Ag Kα radiation *f´* ±0.09, *f´´* ±0.19 were observed. The average refined values deviated by 0.42 for *f´* and 0.86 in case of *f´´* for Mo Kα radiation and by 0.31 for *f´* and 0.44 in case of *f´´* for Ag Kα radiation from the tabulated values according to Sasaki. (1989) As described in S11 the lower refined *f´* values compared to tabulated ones may be attributed to other model deficiencies.  However, it is remarkable that *f´´* is very far off the tabulated value in case of Mo Kα radiation.

## SI References


Brennan, S., Cowan, P. L. (1992). *Rev. Sci. Instrum.* **63**, 850-853

Bourhis, L.J., Dolomanov, O. V., Gildea, R. J., Howard, J. A. K., Puschmann, H. (2015). *Acta Cryst.* A**71**, 59-75

Dolomanov, O. V., Bourhis, L. J., Gildea, R. J., Howard, J. A. K., Puschmann, H. (2009). *J. Appl. Cryst.* **42**

Dyadkin, V., Pattison, P., Dmitriev, V., Chernyshov, D. (2016). *J. Synchrotron Rad.* **23**, 825-829

Henke, B. L., Gullikson, E. M., Davis, J. C. (1993). *At. Data Nucl. Data Tables* **54**, 181-342

Kleemiss, F., Dolomanov, O. V., Bodensteiner, M., Peyerimhoff, N., Midgley, L., Bourhis, L. J., Genoni, A., Malaspina, L. A., Jayatilaka, D., Spencer, J. L., White, F., Grundkötter-Stock, B., Steinhauer, S.,Lentz, D., Puschmann, H., Grabowsky, S. (2021). *Chem. Sci.* **12**, 1675-1692

Meindl, K., Henn, J. (2008). *Acta Cryst.* A**64**, 404-418

Neese, F., Wennmohs F., Becker, U., Riplinger, C. (2020). *J. Chem. Phys.* **152**, 224108

Parsons, S., Wagner, T., Presly, O., Wood. P. A., Cooper, R. (2012) *J. Appl. Cryst.* **45**, 3, 417-429

Ramseshan, S., Abrahams, S. C. (1975). *Anomalous Scattering*

Rigaku Oxford Diffraction (2021). *CrysAlisPro Software System*, Wroclaw, Poland

Sasaki, S. (1989). *KEK Report* **88**, 1-136

Scheinost, C., Claussner, J., Exner, J., Feig, M., Feindeisen, S., Hennig, C., Kvashnina, K. O., Naudet, D., Prieur, D., Rossberg, A., Schmidt, M., Qiu, C., Colomp, P., Cohen, C., Dettona, E., Dyadkin, V., Stumpf, T. (2021). *J. Synchrotron Rad.* **28**, 333-349

Sheldrick, G. M. (2015). *Acta Cryst.* A**71**, 3-8
